# Backbone and methyl assignment of bacteriorhodopsin incorporated into nanodiscs

**DOI:** 10.1007/s10858-019-00289-7

**Published:** 2019-11-21

**Authors:** Laurens Kooijman, Philipp Ansorge, Matthias Schuster, Christian Baumann, Frank Löhr, Simon Jurt, Peter Güntert, Oliver Zerbe

**Affiliations:** 1 Department of Chemistry, University of Zurich, Winterthurerstrasse 190, 8057 Zurich, Switzerland; 2 Institute of Biophysical Chemistry and Center for Biomolecular Magnetic Resonance, Goethe University Frankfurt, Max-von-Laue-Straße 9, 60438 Frankfurt am Main, Germany; 3 Laboratory of Physical Chemistry, ETH Zürich, Vladimir-Prelog-Weg 1–5/10, 8093 Zurich, Switzerland; 4 Department of Chemistry, Tokyo Metropolitan University, 1-1 Minami-Osawa, Hachioji, Tokyo 192-0397 Japan

**Keywords:** Resonance assignment, Membrane protein, Solution-state NMR, Nanodisc

## Abstract

Resonance assignments are challenging for membrane proteins due to the size of the lipid/detergent-protein complex and the presence of line-broadening from conformational exchange. As a consequence, many correlations are missing in the triple-resonance NMR experiments typically used for assignments. Herein, we present an approach in which correlations from these solution-state NMR experiments are supplemented by data from ^13^C unlabeling, single-amino acid type labeling, 4D NOESY data and proximity of moieties to lipids or water in combination with a structure of the protein. These additional data are used to edit the expected peaklists for the automated assignment protocol FLYA, a module of the program package CYANA. We demonstrate application of the protocol to the 262-residue proton pump from archaeal bacteriorhodopsin (bR) in lipid nanodiscs. The lipid-protein assembly is characterized by an overall correlation time of 44 ns. The protocol yielded assignments for 62% of all backbone (H, N, C^α^, C^β^, C′) resonances of bR, corresponding to 74% of all observed backbone spin systems, and 60% of the Ala, Met, Ile (δ1), Leu and Val methyl groups, thus enabling to assign a large fraction of the protein without mutagenesis data. Most missing resonances stem from the extracellular half, likely due intermediate exchange line-broadening. Further analysis revealed that missing information of the amino acid type of the preceding residue is the largest problem, and that 4D NOESY experiments are particularly helpful to compensate for that information loss.

**Electronic supplementary material:**

The online version of this article (10.1007/s10858-019-00289-7) contains supplementary material, which is available to authorized users.

## Introduction

Membrane protein structures are still underrepresented in structure repositories with the number of newly added entries lagging behind expectations (White 2004, 2019), in particular for seven transmembrane (TM) helix proteins. Part of the problem is that the study of these proteins suffers from many biochemical issues (Kim et al. 2009). For solution NMR an additional challenge is the size of the protein-membrane mimetic complex and motions due to the inherent structural instability of these proteins. This leads to poor spectral quality, mainly originating from fast transverse relaxation (Zerbe 2011). Consequently, conventional spectra yield a low amount of correlations, making the assignment procedure significantly more difficult. Nearly complete backbone and significant side chain assignments have only been obtained for a few well-behaving helical membrane proteins, such as sensory rhodopsin (Gautier et al. 2008, 2010), proteorhodopsin (Reckel et al. 2011), and the mitochondrial translocator protein TSPO (Jaremko et al. 2014).

We chose the archaea bacteriorhodopsin (bR) to test our assignment protocol for 7-TM proteins due to its relative ease of expression in bacteria (Nekrasova et al. 2010), a straightforward purification procedure, as well as its possibility to refold upon addition of the cofactor retinal. bR has been studied extensively during the last decades using various biophysical techniques (Birge 1990a, b; Haupts et al. 1999; Ernst et al. 2014; Brown and Ernst 2017), as well as by solution-(Patzelt et al. 2002; Schubert et al. 2002) and solid-state NMR (Harbison et al. 1984a, b; Smith et al. 1984). Moreover, several high-resolution structures are available (Luecke et al. 1999; Schobert et al. 2002; Hasegawa et al. 2018). Schubert et al. (2002) determined assignments by solution-state NMR for bR in dodecylmaltoside (DM) micelles, and could assign approximately one-third of the amide signals.

Etzkorn and coworkers compared spectra of bR in detergent micelles, amphipols and nanodiscs and conclude that detailed studies of bR in nanodiscs should be possible (Etzkorn et al. 2013). In their studies they used cell-free expression and direct artefact-free incorporation into the membrane mimetics, and reported that bR in nanodiscs displays stability superior to when dissolved in DDM micelles. Here, we used the bacterial expression host *E. coli* that permits a range of labeling patterns, such as amino acid-selective backbone and methyl labeling. We incorporated bR into the MSPΔH5 nanodiscs, that were introduced by the Wagner group (Hagn et al. 2013), containing DMPG lipids, allowing us to observe dynamics and structural features in close-to-native environment at temperatures above 0 °C.

**Fig. 1:**
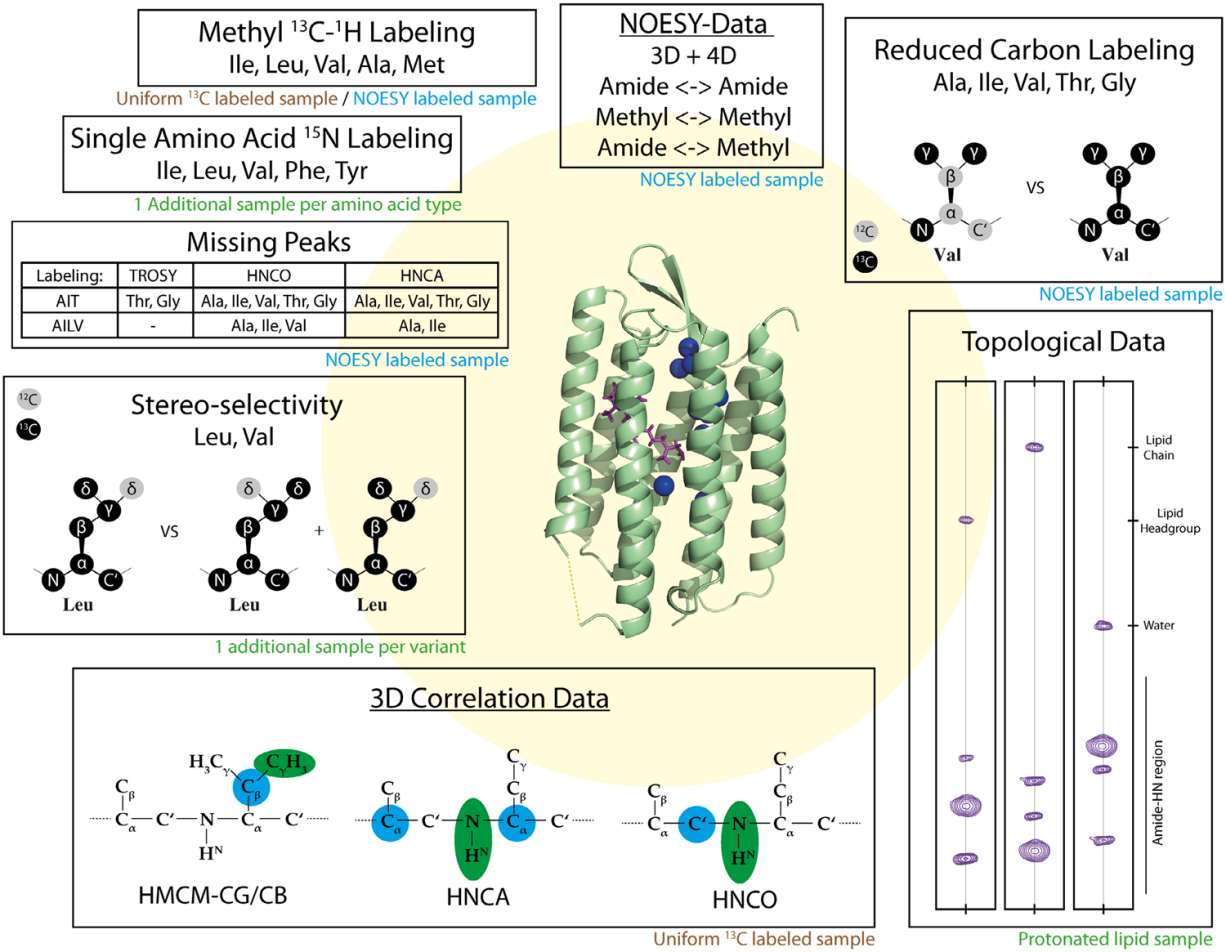
Overview of the seven types of input data that were used in the assignment strategy. For each data set the type of sample is indicated. See text for more details

In this study, we present an assignment procedure for helical membrane proteins and apply it to bR. In the procedure we combine amide- and methyl-derived NMR data from double- and triple-resonance-based NMR experiments with 3D/4D NOESY data, water/lipid accessibility data (Eichmann et al. 2014; Hagn and Wagner 2015), specific biochemical unlabeling resulting from the use of methyl labeling precursors (Kerfah et al. 2015a, b), and amino acid-selective ^15^N labeling (Fig. 1). From a limited amount of samples, we obtained a large set of data that we combined and used as input for the automated assignment procedure FLYA (Schmidt and Güntert 2012) that is part of the CYANA program package (Güntert 2004). Additionally, we analyzed to which extent individual data sources contribute to the overall resonance assignment, and investigated the robustness of our assignment procedure by removing data to mimic a larger and less well-behaving protein. The automated procedure was also able to handle, if not unravel peak duplications due to two isomeric states of the bound cofactor retinal. Our protocol allowed us to assign 62% of all backbone (H, N, C^α^, C^β^, C′) resonances of bR and 60% of the Ala, Met, Ile (δ1), Leu and Val methyl groups. Counting the amide signals revealed that we were unable to observe at least 17% of the protein. To the best of our knowledge, this is the first assignment of an integral membrane protein incorporated into nanodiscs without prior knowledge of assignments from the protein incorporated in detergent micelles.

## Materials and methods

### Protein expression, purification and reconstitution into nanodiscs

For the expression and purification of bR we essentially follow the procedure described by Nekrasova et al. (2010). Therein, C-terminally hexa-His-tagged bO was expressed as a fusion to a N-terminal mistic tag in *E. coli* BL21 (DE3) cells at a temperature of 18 °C for 6 or 20 h (precursor labeling or uniform labeling, respectively). For single amino acid labeling we used the auxotrophic *E. coli* RF18 cells. ILV-labeled bR was produced by addition of α-ketobutyric acid for non-stereospecific ILV methyl labeling (Tugarinov and Kay 2003) or acetolactate for stereospecific methyl-labeling (LeMaster 1989; Kerfah et al. 2015b). Methyl-labeling of Ala, Met and Thr was achieved by addition of the appropriately labeled amino acids.

Mistic-bO was solubilized from inclusion bodies in sarcosyl and urea and the fusion construct was purified by Ni–NTA affinity chromatography. Cleavage of the fusion protein was triggered by addition of thrombin, and mistic and bR were separated by a second Ni–NTA chromatography step. Addition of the membrane scaffolding protein (MSP) MSPΔH5, the lipid 1,2-dimyristoyl-sn-glycero-3-phosphoglycerol (sodium salt) (DMPG), retinal and BioBeads helped to reconstitute bR and incorporate it into nanodiscs. Empty nanodiscs were removed by Ni–NTA chromatography and aggregates and uncleaved mistic-bR by SEC chromatography. For a more detailed description of procedures see the Supplementary Material.

### NMR spectroscopy

All spectra were recorded at a sample temperature of 47 °C on Bruker Av-700, AvIIIHD-800, AvNEO-900 or AvIII-950 spectrometers equipped with cryogenic triple-resonance probes. HNCA and HNCO spectra were measured using programs from the standard Bruker pulse sequence library (Sattler et al. 1999). Three-dimensional HN(CA)CB and HN(COCA)CB experiments were carried out at 800 MHz ^1^H Larmor frequency and employed [^15^N,^1^H]-BEST-TROSY type pulse sequences (Solyom et al. 2013). All experiments utilized ^2^H decoupling during ^13^C evolution times or ^13^C^α^–^13^C^β^ transfer steps. Measurement times were approximately 2 days for the HNCA and HNCO experiments and 5 days for HN(CA)CB and HN(COCA)CB. Four-dimensional ^13^C, ^13^C- and ^13^C, ^15^N-separated NOESY spectra were recorded at 900 and 950 MHz ^1^H frequency, respectively, with a mixing time of 250 ms. Pulse schemes were adapted from 3D SOFAST HMQC-NOESY-HMQC sequences (Rossi et al. 2016) by inserting proton evolution times into the SOFAST-HMQC (Schanda et al. 2005) modules preceding the NOE mixing periods. Both experiments employed non-uniform sampling and were processed with the compressed sensing algorithm in TopSpin 3.5. For the 4D ^13^C-SOFAST-HMQC-NOESY-^13^C-SOFAST-HMQC 21% (ILV) and 11.6% (AILV) of the full time-domain data grid was acquired in a total measurement time of 13.5 d (ILV) and 14 d (AILV), while the sparseness of the 4D ^13^C-SOFAST-HMQC-NOESY-^15^N-SOFAST-HMQC was 17.2% (ILV) and 11.1% (AILV) (20.5 days and 16 days total measurement time, respectively). Four and 16 transients per FID were accumulated for HCCH– and HCNH-NOESY experiments, using relaxation delays of 0.5 and 0.7 s, respectively. Acquisition times were 53.2 ms (*t*_3_), 31.3 ms (*t*_2_, ^15^N) and 9 ms (*t*_1_, ^13^C) for the HN(CA)CB; 53.2 ms (*t*_3_), 29.9 ms (*t*_2_, ^15^N) and 5.2 ms (*t*_1_, ^13^C) for the HN(COCA)CB; 66.5 ms (*t*_4_), 22.4 ms (*t*_3_, ^13^C), 14.9 ms (*t*_2_, ^13^C) and 16.1 ms (*t*_1_, ^1^H) for the HCCH 4D NOESY with ILV labeling; 66.5 ms (*t*_4_), 22.5 ms (*t*_3_, ^13^C), 22.5 ms (*t*_2_, ^13^C) and 17.1 ms (*t*_1_, ^1^H) for the HCCH 4D NOESY with AILV labeling; 33.6 ms (*t*_4_), 17.6 ms (*t*_3_, ^15^N), 13.4 ms (*t*_2_, ^13^C) and 15.2 ms (*t*_1_, ^1^H) for the HNCH 4D NOESY with ILV labeling and 33.6 ms (*t*_4_), 17.6 ms (*t*_3_, ^15^N), 14.0 ms (*t*_2_, ^13^C) and 16.1 ms (*t*_1_, ^1^H) for the HNCH 4D NOESY with AILV labeling. 4D NOESYs were processed to 512 × 192 × 128 × 64 (HCCH-ILV), 512 × 192 × 192 × 80 (HCCH-AILV), 512 × 88 × 128 × 6 4 (HCNH-ILV) and 512 × 88 × 128 × 80 (HCNH-AILV) data points using cosine-shifted sine bells for data apodization. A summary of details for all experiments is provided in Table S1.

### FLYA calculations

All runs of the FLYA algorithm were performed using 20,000 iterations of local optimization, a population size of 250, and 40 independent runs. The chemical shift tolerance was set to 0.03, 0.4 and 0.4 ppm for ^1^H, ^15^N and ^13^C, respectively. These conditions were optimized to yield a high amount of strongly assigned atoms/residues. Residues are indicated as correctly assigned in Fig. 6 below where the FLYA output shows both amide or methyl atoms as strongly assigned (i.e. 80% or more of the 40 independent runs yielded, within the aforementioned tolerances, the same chemical shift value) and the chemical shifts were within the same tolerances of the manual assignments.

Additional data types were incorporated into FLYA by adding additional ^15^N-HSQC or ^13^C-HSQC peaklists, which contain only the anchor signals of the spin systems that show a specific characteristic. The corresponding expected peaklists were generated by FLYA using the sequence for single amino acid labeling and backbone unlabeling data types. For the topology data we extracted distance restraints from the 1M0L bR crystal structure that had been incorporated into a nanodisc, in silico, using an online molecular dynamics (MD) input generator tool, CHARMM-GUI (Lee et al. 2006, 2016) and running a short MD simulation using Gromacs (Berendsen et al. 1995). We extracted multiple lists of residues with distance cutoffs between 4 and 10 Å using Pymol and let FLYA generate the expected peaklist using these residue numbers. For all expected peaklists that were determined using a distance cutoff, i.e. NOESY and topology data, we optimized the cutoff such that the resulting expected peaklist contained ~ 10% more peaks than the measured peaklist. One exception was the proximity to the lipids, which were unexpectedly observed also for amide moieties on the inside of the helix bundle (Fig. S16) and required a higher distance cutoff for generating expected peak list. The cutoff we chose was 8 Å as opposed to 6 Å, which we used to indicate proximity to water.

To analyze the FLYA output we used a spin system matching procedure over the 40 independent runs that started with different seeds. The standard FLYA procedure is to consolidate single atom assignments from these runs, yielding ‘strong’ assignments when in more than 80% of the runs the chemical shift is within a given tolerance. We modified the procedure to do this for entire spin systems, which include all observed chemical shifts of H, N^H^, C^α^, C^β^, C_−1_′, C_−1_^α^, C_−1_^β^ and C′. The matching is done for the complete spin system, or only for amide H/N^H^ atoms. We ranked a match as reliable when each individual atom within the spin system is also marked as ‘strong’ by the single atom consolidation of FLYA. A match that is not unique or comprises only part of the residue, i.e. only intraresidual or H/N^H^ atoms, is a sign of ambiguity in the data, and we treated these matches with more caution. Final assignments were checked manually for the presence of sequential correlations (for the backbone) and NOE contacts in the 4D NOESYs (for methyls). The amount of unique and reliable matches also proved to be a good indicator for the performance of the FLYA assignment. Scripts for the matching procedure can be requested from the corresponding author.

Further materials and methods are in the SI.

## Results

### Expression

Bacterioopsin (bO), the retinal-free form of bR, was expressed as a fusion to mistic (Roosild et al. 2005) in the *E. coli* BL21(DE3) strain (Nekrasova et al. 2010). Inclusion bodies were solubilized in a mixture of urea and sarcosyl. After thrombin cleavage, Ni–NTA affinity chromatography allowed removal of mistic and exchange into SDS detergent. Folded bR was obtained by subsequent addition of retinal and incorporation into nanodiscs by adding biobeads to the SDS/DMPG/MSPΔH5 mixture (Supplementary Material).

**Fig. 2:**
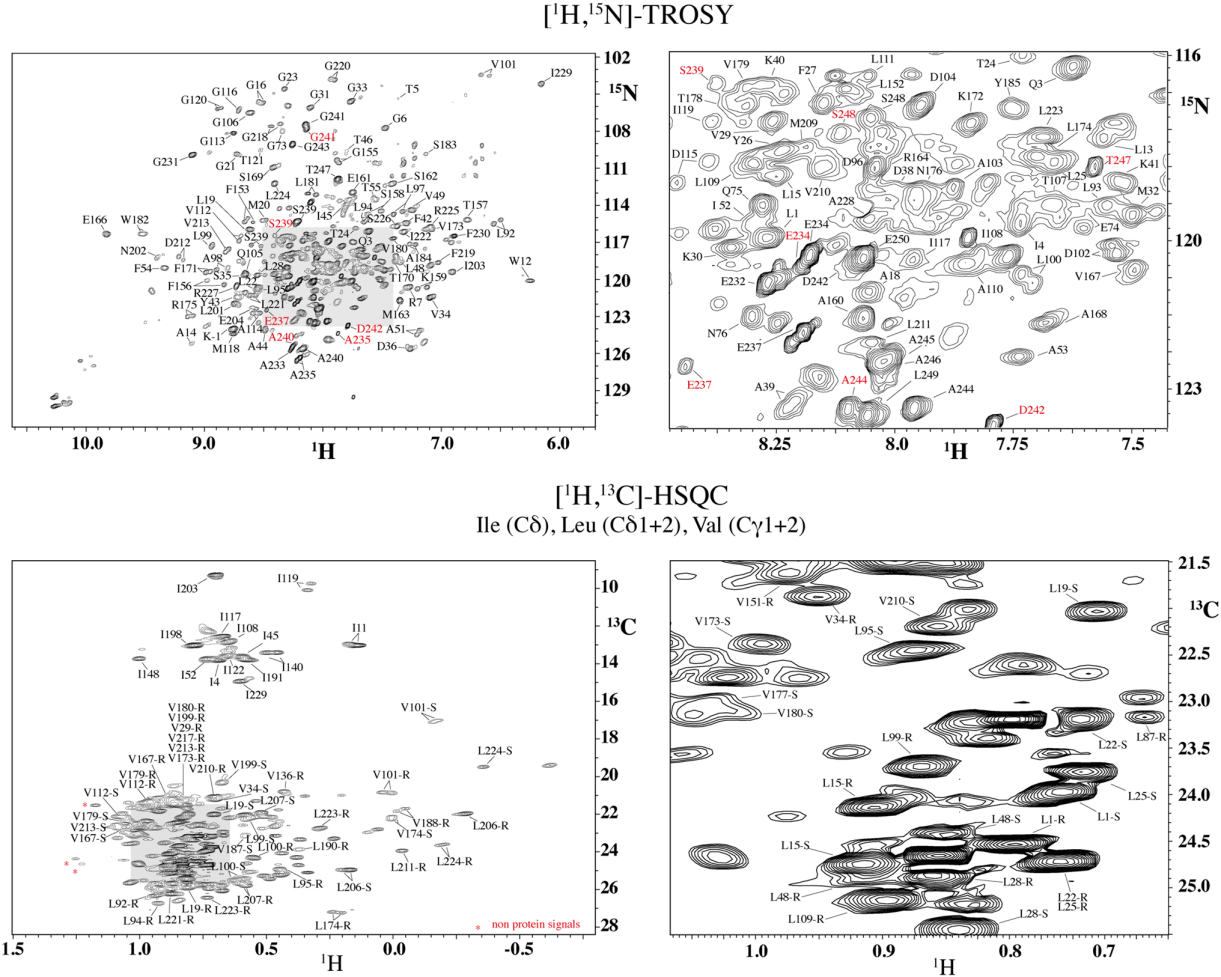
2D fingerprint spectra. 2D [^15^N,^1^H]-TROSY (top) and [^13^C,^1^H]-HSQC (bottom) spectra of approx. 445 μM [^2^H,^15^N,^13^C, Leu:^1^Hδ_1/2_, Val:^1^Hγ_1/2_, Ile:^1^Hδ_1_]-bR (Sample 7, Table S2), *T* = 320 K, measured at 900 MHz ^1^H frequency. The grey-shaded areas in the overview spectra on the left are enlarged on the right. Peaks marked by red assignments are from the minor species in the C-terminus, and peaks that do not originate from the protein are marked by red stars in the [^13^C,^1^H]-HSQC

For NMR measurements, we produced samples with different combinations of isotope labeling, including methyl group [^13^C,^1^H], side chain [^12^C,^2^H], perdeuteration, and single amino acid type ^15^N (Table S2). Utilizing specifically labeled metabolic precursors introduced by the groups of Kay (Tugarinov and Kay 2003) or Boisbouvier (LeMaster 1989; Kerfah et al. 2015b), we obtained [^1^H,^13^C] methyl labeling for Ala, Ile, Leu, Val and Met residues with stereo-selective and non-stereo-selective incorporation for Leu and Val residues (Fig. 2). In principle, the density of labeled methyl groups is higher in the non-stereo-selectively labeled variant, but labeling at each position is reduced by 50%, making such samples problematic for NOESY experiments (Gans et al. 2010; Kerfah et al. 2015a, b). These precursors also allowed for ^13^C labeling of side-chain carbon atoms for assignment purposes, or ^12^C labeling for increased sensitivity in methyl NOESY experiments (Gans et al. 2010).

Amino acid type-selective labeling with ^15^N was obtained using the auxotrophic *E. coli* strain RF18, in which many de novo amino acid synthesis pathways have been disabled (Lin et al. 2011, 2015). Therefore, the amino acids supplemented to the growth medium allow for labeling of individual amino acid types. The RF18 strain is optimized to minimize scrambling for Tyr, Phe, Ile, Leu and Val, so we expressed bR using single amino acid type ^15^N labeling for these residues (Fig. S8). We decided against perdeuteration in these expressions, because the spectral quality of protonated bR was sufficient to identify the single amino acid type signals. Isotope scrambling of ^15^N was only visible for the very intense peaks originating from the C-terminus. Since these signals were common in all amino acid-selectively labeled samples they were identified comparably easily.

### Triple-resonance data

**Fig. 3:**
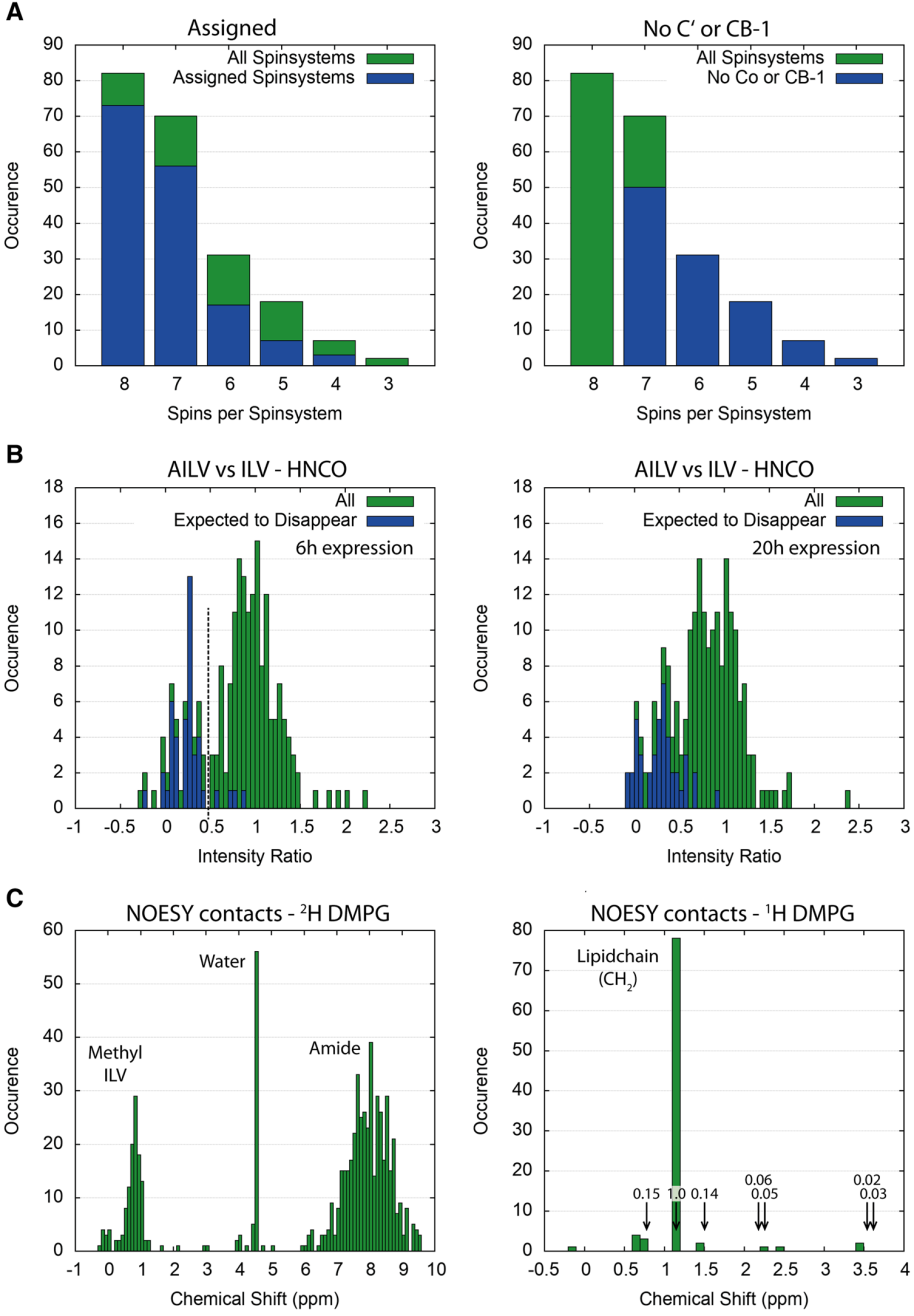
Occurrence of correlations and proximity. **a** Histograms of the number of spin systems with 3–8 triple resonance backbone correlations (green), overlaid in blue with the number of assigned spin systems (left) or the number of spin systems that lack the C′ or C_–1_^β^ correlation (right). **b** Histograms of the peak intensity ratios between fully backbone-labeled (ILV) and partially unlabeled (AILV) samples with 6 h (left) and 20 h (right) expression. Assigned signals that were expected to disappear in the AILV labeled samples are shown in blue. The cutoff chosen to identify the missing signals is marked with a dashed line. Detailed data from unlabeling is given in Fig. S12. **c** Histograms of the number of ^1^H cross peaks at given chemical shift values observed in ^15^N-resolved NOESY recorded with ILV methyl labeling and deuterated lipids (left), and an expansion of the methyl region recorded with perdeuterated methyl groups and protonated lipids (right). The arrows and numbers in the histogram indicate the position and relative intensity of the lipid signals observed in a 1D proton spectrum

Traditional protein backbone assignment starts with describing amide-anchored spin systems by intra-residual and sequential correlations. For large proteins or protein complexes 3D correlation spectra lack many of these correlations, even in their TROSY-based versions (Pervushin et al. 1997; Salzmann et al. 1998). The size of the bR-ND complex is estimated to be ~ 127 kDa from SEC-MALS data (Hagn et al. 2013), which is in agreement with the correlation time of 44 ns measured at 320 K using TRACT (Lee et al. 2006) (Fig. S9). To unravel peak clusters in the [^1^H,^15^N]-TROSY spectrum we used the HNCO spectrum to determine exact amide peak locations. Nevertheless, we could detect the C_–1_^β^ and C′ correlations only in 60% and 68% of our spin systems, respectively, with only 39% showing all 6 possible correlations (Fig. 3a, Table S3). Additionally, 34% of the observed spin systems contain only one sequential contact (C_−1_^α^, C_−1_^β^ or C′), and 8% miss two. For the methyl groups we measured the correlation to C^γ^, C^β^ and C^α^ for Ile, Leu and Val (Tugarinov and Kay 2003). Due to low signal dispersion not all of the methyl correlations could be distinguished unambiguously, but for all spin systems we were able to determine the amino acid type.

In the triple resonance data, peak doubling occurred for many residues. We could verify peak duplications reported before (Schubert et al. 2002) and identified many more. In addition, we observed a minor second conformation in the C-terminus (Table S4), which originates either from proline cis/trans isomerization or from Asp/isoAsp-Gly isomerization of Asp242/Gly243 (Tugarinov et al. 2002; Grassi et al. 2017). The proline isomerization was detectable from sequential connections to proline, and the presence of the isoAsp modification was implied by the lack of sequential correlations between isoAsp-Gly, despite intense peaks for these residues, and the delayed appearance of isoAsp in spectra, which isomerizes over the course of days at low temperatures. We removed spin systems related to the minor state from the assignment procedure. However, we would like to point out that these signals had little effect on the automated assignment. The third source of doubling occurs throughout the whole sequence of bR and is visible as small chemical shift differences in the ^15^N-TROSY and ^13^C-HSQC spectra (Fig. S10). We identified this doubling as corresponding to the two isomeric retinal states present after dark adaptation (manuscript in preparation), which are all-trans and 13-cis/15-syn retinal (Harbison et al. 1984b), with the light-activated state of retinal being 13-cis/15-anti. The interconversion process is very slow on the NMR time scale and all triple-resonance correlations are almost identical in both states, making the doublets easily identifiable (Table S5). To ease automated assignment, we retained only one of the two sets of peaks in the assignment procedure and analyses. However, we cannot safely exclude that a few peak duplicates remain in overlapping regions of the data set.

### NOESY data

To supplement the triple-resonance experiments, we measured multiple 3D and 4D NOESY experiments. Our goal was to evaluate the relative merits of 3D vs. 4D experiments. For example, we measured the 4D methyl-to-methyl NOESY (HC–CH) together with two 3D NOESYs with either ^1^H or ^13^C in F1 (Hc–CH and hC–CH, respectively). We expected to observe more cross peaks in the 3D experiments, but their amount is actually similar in 3D and 4D spectra (Table S6), and the assignment procedure clearly worked better with the 4D experiments (see Fig. 6 below). In addition, we measured the ^15^N component of the amide-to-amide connections with a ^15^N-resolved NOESY in which the ^15^N chemical shift was labeled in F1 (hN-NH). Compared to the standard ^15^N-resolved NOESY (H-NH), the pulse sequence contains an extra INEPT step and we observed 78% of the connections observed in the H-NH NOESY (Table S6). A similar experiment was used by Schubert et al. (2002) in the previous assignment of bR in micelles.

The 4D NOESY spectra were measured using racemic (ILV) and stereo-selective (AILV, proR for Leu/Val) methyl-labeled samples. The non-stereospecific labeling includes labeling of both methyl groups in Leu and Val, but, as only one of them is labeled at a time, the intensities of NOE cross peaks between them are reduced to 25%. Both labeling types yielded a similar amount of observed cross peaks corresponding to a similar average distance (Fig. S11a, green bars). To compare both labeling types, we removed all proS-related NOESY correlations from ILV and Ala-related moieties from the AILV dataset. We found that the stereo-selective labeling yielded significantly more proR-related connections (Fig. S11b). The average distance for these connections is comparable to the other connections (Fig. S11a, blue bars). This indicates that stereo-selective labeling does not result in NOE connections with longer distance, but instead displays more transient connections. Therefore each 4D NOESY experiment brings complementary NOE connections that improve the assignment. We suspect that 4D NOESY spectra are particularly helpful and important for helical membrane proteins, for which chemical shift dispersion is small and knowledge of just the proton *or* carbon frequency from the NOE-related spin in the corresponding 3D spectra is often not sufficient to unambiguously identify the residue.

### Additional spin system information

To compensate for the reduced spectral quality in bR, compared to soluble proteins, we looked for other sources of information that could be measured easily, ideally without preparing additional samples. We exploited the unlabeling of side chain carbon and amide nitrogen atoms resulting from NOESY-optimized precursors for methyl labeling (Kerfah et al. 2015a) (Table S2), measured water/lipid accessibility restraints with NOESY and H/D-exchange, and performed single amino acid type ^15^N labeling (Fig. S8). These methods use the most sensitive experiments, i.e. 2D ^15^N-TROSY, 3D HNCO, and HNCA, which are also applicable to larger and less well-behaved systems. The additional data contribute to the assignment procedure by restricting the possibilities for sequential contacts and amino acid type.

To identify unlabeled positions, we analyzed intensity ratios in ^15^N-TROSY, HNCO, and HNCA spectra derived from samples with and without specific unlabeling of the backbone (Figs. 3b and S12). Short expression times were necessary to avoid scrambling by *de*-*novo* amino acid synthesis and to obtain a clear-cut difference between labeled and unlabeled positions (Fig. 3b). One of the main problems for identifying disappearing peaks is the overlap of signals. To avoid false assignments, we only used the peaks that were significantly decreased (Fig. S12). In AILV-labeled bR we identified 54 out of 65 expected missing correlations in the HNCO spectrum, and 34 out of 44 in the HNCA spectrum. For AIT-labeled bR, we identified 59 out of 89 expected missing correlations in HNCO, and 35 out of 44 in [^15^N,^1^H]-TROSY.

With a reasonably good structure or model at hand, one can predict proximity of amide and methyl groups to lipid or water molecules. From ^15^N- or ^13^C-resolved NOESY spectra we were able to identify contacts to water and lipids (Fig. 3c) in addition to the disappearance of ^15^N-TROSY peaks after H/D-exchange (Fig. S13). The ^15^N-resolved NOESY and H/D-exchange complemented each other very well in crowded spectral regions. The deuterium exchange helped to recognize whether all or only part of the overlapping amides were close to water, while the NOESY revealed proximity to internal water molecules. Overall, we identified 137 amide spin systems that are close to water.

To detect proximity to lipids, we incorporated bR into nanodiscs containing protonated DMPG, measured ^15^N- and ^13^C-resolved NOESY spectra, and compared to spectra recorded with perdeuterated lipids (d_54_-DMPG, only the central glycerol is protonated). The majority of the extra peaks in the sample with protonated lipids occur at 1.17 ppm (Fig. 3c), which corresponds to the methylene protons in the acyl chains. Other signals could be observed too, but were too few to be reliably used as restraints. To exclude signals from protein methyl resonances, we used a non-methyl labeled sample. We identified 73 amide and 72 methyl spin systems that are in proximity to the lipid acyl chain.

### Automated assignment procedure

Given all these data types that need to be considered during the assignment procedure, a manual approach presents a formidable challenge. Therefore we decided to use the automated assignment algorithm FLYA (Schmidt and Güntert 2012), which is part of the program CYANA (Güntert 2004). FLYA can easily incorporate all of the above-mentioned data into an efficient and automated resonance assignment procedure, even for large systems with complex input data (Gauto et al. 2019). In addition, we analyzed the performance of the FLYA assignment procedure when supplying different subsets of the input data.

In short, the FLYA algorithm generates expected peaks for each spectrum type used and matches these to the measured peaks. Each peak in the expected peak lists contains assignment information, but the corresponding chemical shift positions are initially unknown and can only be estimated roughly at the start. For each spectrum, the expected and measured peak lists are matched in multiple iterations, thereby connecting assignments with experimental chemical shifts. From all possible assignments the algorithm leads to one solution that best reflects the measured data, i.e. produces the highest score (Schmidt and Güntert 2012). This procedure is repeated multiple times with different random number generator seed values for the matching algorithm, and assignments that are the same in more than 80% of the repeats are classified as “strong”. Strong FLYA assignments have been shown to be much more reliable than other “weak” assignments (Schmidt and Güntert 2012). The assignment result is reported for each individual atom (Fig. S14).

The generation of realistic expected peak lists is a crucial step of FLYA. Without the correct expected peaks certain assignments could be missed, especially for a sparsely defined system. Nevertheless, it has been shown that the assignment procedure remains reliable when the experimental peak lists lack significant amounts of data and/or contain noise peaks (Schmidt and Güntert 2012). For through-bond spectra (e.g. HNCO, HNCA, etc.), the generation of expected peaks is straightforward as the sequence provides unambiguous expectations. For NOESY data the expected peaks are distance-dependent, and hence require a structural model. We have generated structure-based expected peaks using a distance cutoff chosen such as to yield at least 10% more expected than measured peaks in order to account for ambiguity in our NOESY data. We also reduced the observation probability for expected NOESY cross peaks corresponding to distances of 0.5 and 1.0 Å below the distance cutoff by a factor of 0.8 and 0.9, respectively. The observation probability is used in the scoring algorithm to guide the assignments and our goal was to reduce the penalty for missing these long-range contacts.

For the structural model we used the 1.47 Å resolution crystal structure, 1M0L (Schobert et al. 2002). However, we saw very little difference in backbone assignments when using a 2.3 Å resolution structure, 1BRR (Essen et al. 1998). In fact, we expect that even homology models can supply enough structural information when using this assignment procedure for 7-TM proteins. When no template structure is provided, a series of randomized structures is generated by FLYA from which only short-range NOE contacts can be expected. For bR, we observed that partial backbone assignments are still possible, but the methyl group assignment was heavily impaired (data not shown). In its original protocol FLYA is used in combination with structure calculations, and a self-consistent set of assignments, resulting in a good structure, validates the latter. Since in our case, based on the scarcity of NOEs, no structure can be calculated, such verification is impossible, and hence it is even more important to check the correctness of assignments (vide infra).

To assess the expectations for the water and lipid proximity, the distance of each amide and methyl moiety to the closest water and lipid acyl chain were extracted from a model bR-nanodisc complex structure (see Materials and Methods). We started with a distance cutoff that produced ~ 10% more expected than measured peaks, but preliminary assignments revealed that many expected peaks were missing, mainly those that represent lipid proximity. Therefore, we increased the distance cutoff such as to produce about three times more expected than measured peaks in the lipid and water proximity lists.

To obtain spin system assignments after the FLYA procedure we developed a spin system matching procedure that matches the single atom output to our measured data. The FLYA output (Fig. S14) provides chemical shift information for individual atoms, disregarding the original spin systems. Each atom that is assigned to at least one peak appears with a chemical shift value in the FLYA assignment. However, assignments that are not supported by unequivocal experimental data will not be marked as ‘strong’ as they lack self-consistency over individual runs within the FLYA algorithm. To further increase the reliability of the assignment, our post-FLYA matching procedure considers the sets of backbone atoms of each measured spin system, and matches these to the single-atom chemical shifts of the FLYA output. From the FLYA assignment with our complete dataset we obtained 140 single significant matches to complete backbone spin systems from which we could confirm 130 as correctly assigned using sequential connections. The ten matches we could not confirm are mainly based on 4D NOESY connections that we deemed too ambiguous to fix the assignment. Additional partial matches or spin systems that match to multiple residues may indicate overlap or wrong peak picking and could still be used, but required more caution. From the 20 partial matches, 9 could be confirmed and 3 were erroneously assigned. Additionally, FLYA was not able to make 16 assignments that we established manually. The majority of these assignments are located in the N-terminus, a leucine repeat, and an alanine triplet in the C-terminus. The repeats are difficult to assign due to overlap, and the N-terminus shows conflicting NOESY connections to water and lipids. Interestingly, using restricted input data, FLYA was able to find some of these assignments. We also found that the amount of unique and significant spin system matches is a useful and reliable statistic to optimize the assignment procedure. Due to overlap and lack of dispersed scalar couplings the same method is unfortunately not as useful for methyl data.

**Fig. 4:**
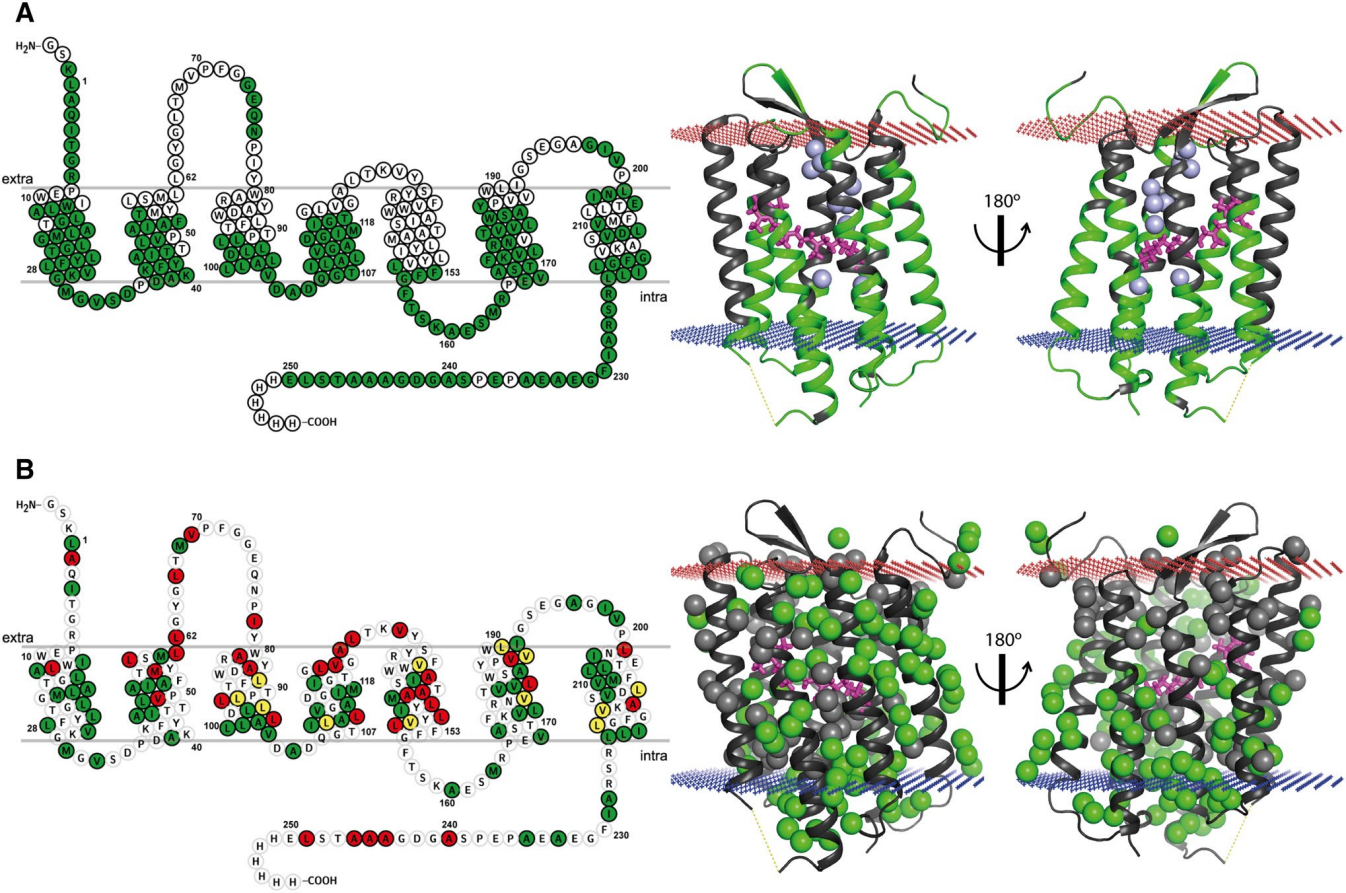
Overview of the chemical shift assignments of bR. **a** Residues with assigned backbone resonances are shown in green on the snakeplot and the structure. **b** Assigned methyl groups of Ala, Ile(δ1), Leu, Val, Met are shown on the structure in green, where each sphere indicates the methyl carbon. On the snakeplot, the color indicates whether all methyl groups (green), only one of the prochiral ones (yellow), or no methyl group (red) is assigned. Unassigned backbone amide and methyl groups are colored in grey on the structure. The membrane position is depicted by red (extracellular) and blue (intracellular) crosses, internal water atoms as light-blue spheres (only in A) and the retinal with magenta sticks. The crystal structure (1M0L) lacks coordinates for residues 156–162 (marked by a dashed yellow line), which have been assigned completely. The N-terminus up to Thr-5 has been added in an arbitrary conformation

To be able to report on the efficiency of the automated assignment method and the relative importance of different data types it was essential to confirm the accuracy of the procedure. We verified the backbone assignments by manually checking the sequential connections using the triple-resonance and ^15^N-resolved NOESY spectra. Where clear-cut sequential correlations are observed, and the C^α^/C^β^ chemical shifts matched the correct amino acid type, we accepted the assignments. With this procedure it is difficult to confirm strongly assigned stretches of less than 3 residues, and therefore some strong assignments (Fig. S14) were discarded. For the methyl assignments, we compared correlations from the 4D methyl-to-methyl and methyl-to-amide NOESY spectra to expectations from the structural model. Additional methyl assignments were possible by manually inspecting 4D connections that had not been assigned by FLYA. Overall, we assigned 156 amide groups (62%) and 103 methyl groups (60% of Ala, Ile(δ1), Leu(δ1/2), Val(γ1/2), Met) (Fig. 4).

### Assessment of assignments and restraints

**Fig. 5:**
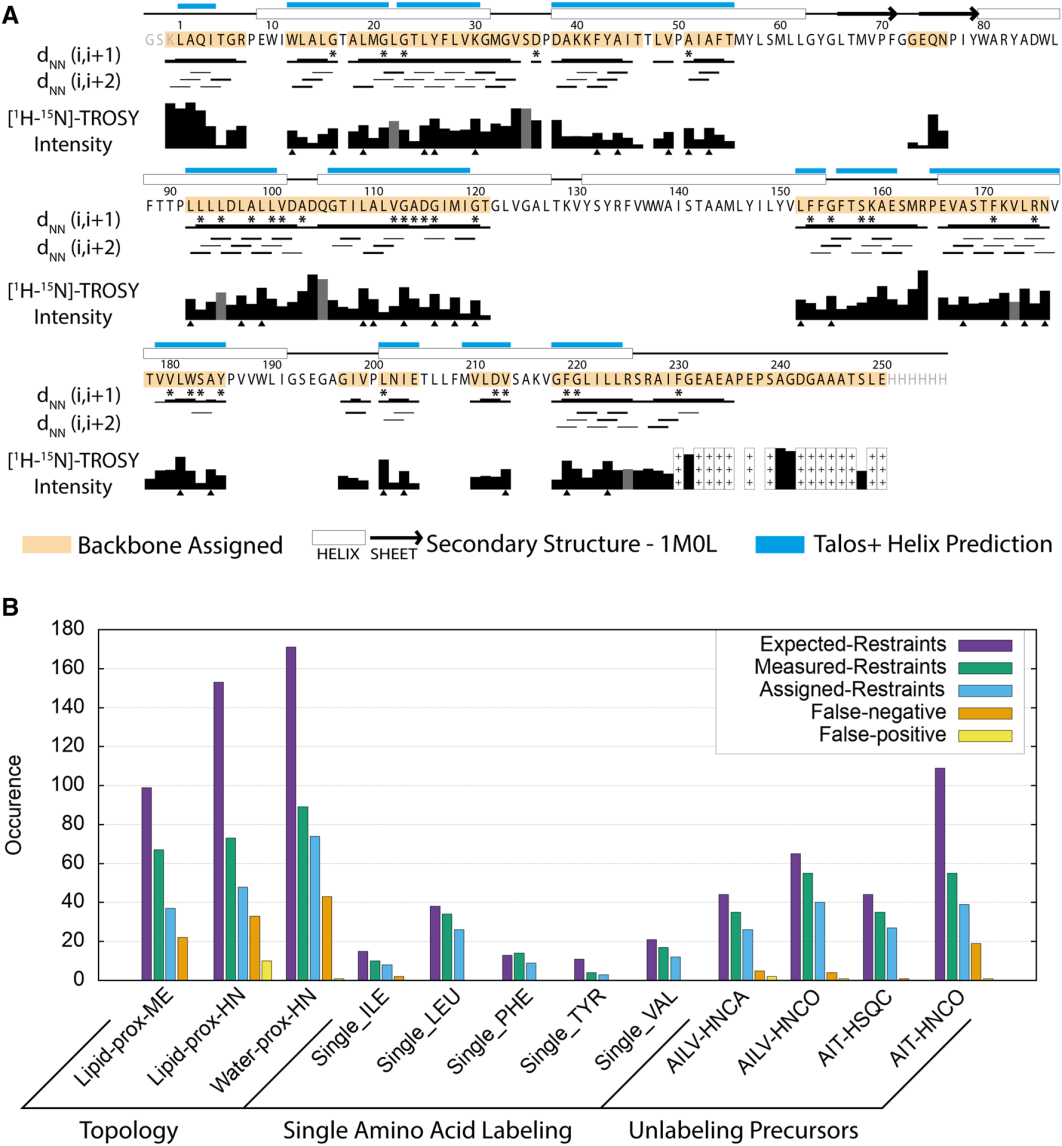
Assessment of assignments and restraints. **a** Overview of the assignment, presence of sequential NOE contacts, TALOS + prediction for helices, and peak intensities in the [^15^N,^1^H]-TROSY spectrum. The thickness of the bars for short-range NOEs indicates how often they are observed with a maximum of 4 for *i*, *i* + 1 and 2 for *i*, *i* + 2. Both HNH and hNNH NOESY contacts were combined to resolve overlap. Gray bars for [^15^N,^1^H]-TROSY intensities indicate overlapping spin systems for which the intensity could not be attributed to a single residue. Intensities at the C terminus are much stronger and denoted by “+++”. Residues with peak doubling from dark-adaptation in the [^15^N,^1^H]-TROSY are marked with a star and the corresponding peak intensity is the sum of both peaks. The residues with relatively strong TROSY peaks are marked with a triangle (Fig. S15). **b** For each additional data type, the number of expected restraints (violet), used to create expected peaklists, the number of observed restraints (green), added as input to FLYA, and the amount of restraints that are part of an assigned spin system (cyan). Expected but not observed (false negative; orange) or observed but not expected (false positives; yellow) restraints indicate disagreements between measurement and model

With chemical shift assignments of bR at hand we analyzed how the consistency of the chemical shifts agree with the secondary structure from the crystal structure, and we reviewed the usefulness of additional data types (Fig. 5a). TALOS + predictions (Shen et al. 2009) revealed that the helix locations largely agree with the positions in the crystal structure. The N terminus and residues 156–162 are not part of the reference structure whereas our data clearly indicate that these parts are both helical, as also seen in a previous study (Schubert et al. 2002). These observations are supported by the presence of sequential NOEs within the helices, which are absent in the loops. Peak intensities in the [^15^N,^1^H]-TROSY reflect the flexibility of the backbone. We observe strong peaks for the loops and termini, in particular for residues at the C-terminus. While in general signals from residues within the membrane are weaker, we observed patterns of relatively intense signals (Fig. 5a, triangles). These more flexible amide moieties are on the outside of the helix bundle in helices D and F. Interestingly, intense peaks were also observed on the inside of helix A pointing towards water molecules (Fig. S15). Moreover, most of the intense peaks are next to or in the vicinity of Gly and Pro residues. These observations can be used as a proxy for backbone dynamics as low signal intensities preclude performing conventional relaxation experiments (Solt et al. 2017).

To assess the quality and usefulness of the additional data types we compared the number of experimental assignment restraints obtained from the additional data types with their expected number, where each assignment restraint is a single observation of lipid/water proximity (topology), single amino acid signal or a missing peak (unlabeling precursors) (Fig. 5b). Additionally, we counted the assigned spin systems that were expected to have a restraint without observing one (false negative) and those which contained a restraint that was not expected (false positive). In general, we see more false negatives than false positives, which we attribute mainly to peak overlap. As we only included positive identifications, we inspected the false positives more closely. In the unlabeling data false positives arose exclusively from very weak peaks for which the intensity ratio is strongly affected by noise. In the lipid proximity data false positives arose from the fact that the N-terminal residues form contacts with the lipid acyl chain (Fig. S16), which is unexpected from the model.

The proximity to water and lipid data followed expectations with some exceptions (Fig. S16). Residues close to water are mainly located in loops, and lipid contacts are observed in the membrane region. Interestingly, lipid contacts are observed over the entire membrane range, including the headgroup region, and also for amide moieties inside the helix bundle. Since each NOE represents a contact to a lipid chain methylene, this indicates flexibility within the helix bundle. We also observed that the N-terminus is in contact with lipids as well as with water. We suspect this to be specific for nanodiscs as these residues are the only ones with significantly different chemical shifts compared to a previous partial assignment of bR in DM micelles (Schubert et al. 2002). Water proximity is observed not only for loop residues, but also for three amide moieties inside the helix bundle, namely Val49, Asp212 and Val213. Multiple amide moieties close to internal water molecules in the structure show no or only very weak NOEs, indicating that these water contacts are relatively short lived. Interestingly, two other residues, Trp182 and Gly220, display NOE cross peaks at 0.15 ppm upfield of the bulk water chemical shift. They are both from the intracellular side of bR where two water molecules are separated from bulk water by a large hydrophobic barrier.

### Influence of additional data

**Fig. 6:**
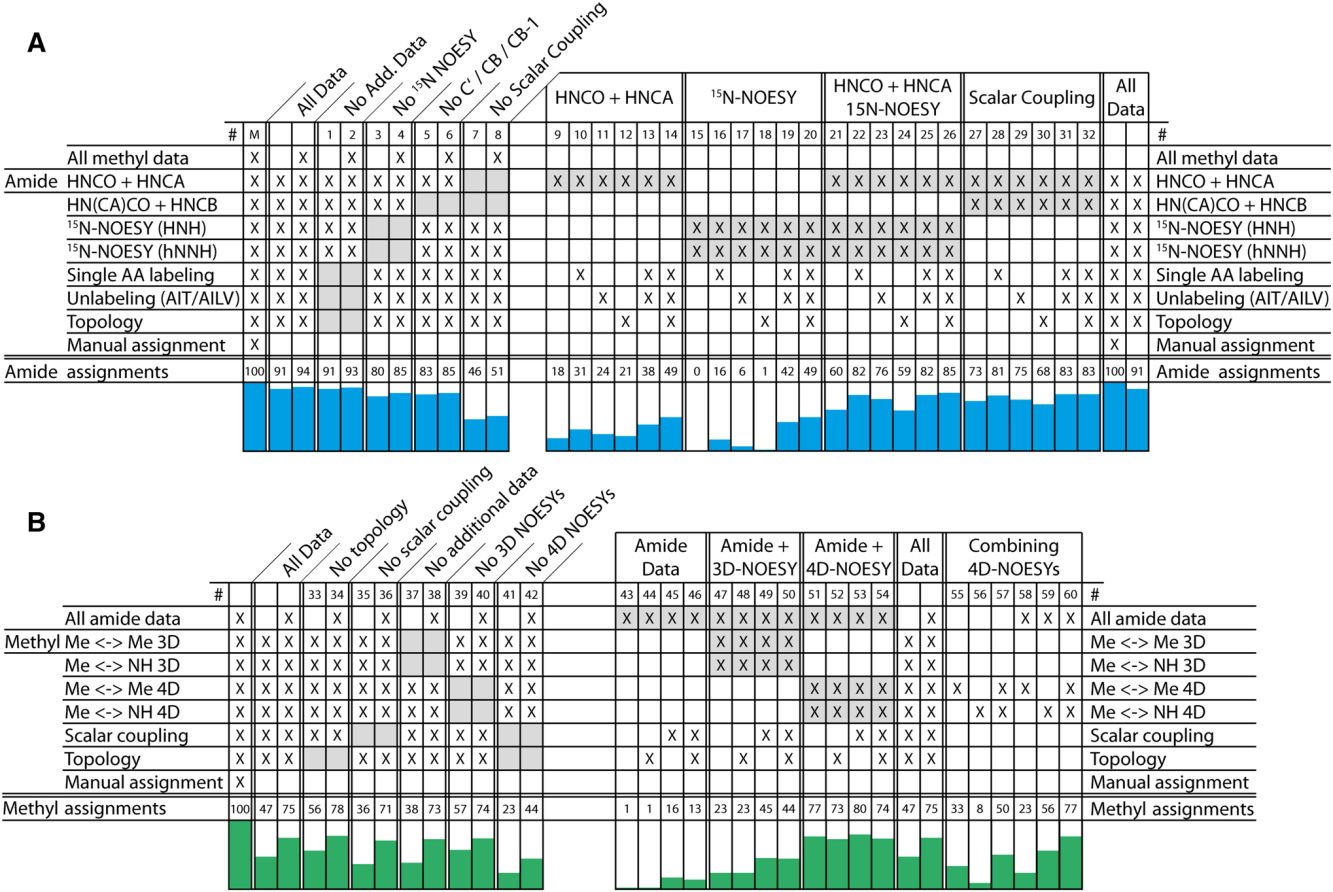
Impact of different data on FLYA assignments. The amount of correct amide (**a**) and methyl (**b**) assignments are shown after performing otherwise identical FLYA runs with different input data. Each column represents a single assignment run with each ‘X’ marking data that were used as input. The amount of assignments is given in percent compared to the manual assignment. Assignment runs where data were removed from a full dataset (alternating between only methyl/amide data and both) are shown in columns labeled 1–8 and 33–42 with the excluded data highlighted in grey and labeled on top of the plot. The influence of the additional data types was probed using different basis sets, highlighted in grey in columns 9–32 and 43–54 and labeled on top of the plot. Columns 55–60 show the combination of methyl-to-methyl and methyl-to-amide 4D NOESY spectra, with and without amide data. The [^15^N,^1^H]-TROSY, [^13^C,^1^H]-HSQC and, in the case of methyl data, identification of the type of amino acid and their stereo-specific annotation was included in all calculations

The main aim of this study was to investigate the effectiveness and reliability of our automated assignment procedure for large membrane proteins. For this purpose, we performed FLYA runs with reduced amounts of data and monitored the extent to which FLYA could make correct and reliable assignments. In a first step, we removed single data sources (Fig. 6, columns 1–8 and 33–42), which revealed that removing the additional data types has no or limited impact on both backbone and methyl assignments (columns 1–2 and 33–34). In addition, removing either ^15^N-resolved NOESY or C^β^/C′ correlations results in a larger effect, but still allows assigning 80% and 83% of the manual assignments, respectively (columns 3 and 5). We suspect that the remaining data contain redundant information that compensates for the excluded data. In fact, 46% of the manual backbone assignments are still made when excluding all scalar couplings (column 7). The biggest reduction in methyl assignments is found when the 4D NOESY data is removed. However, with the inclusion of amide data still 44% of the manual methyl assignments are made (columns 41–42).

To reduce the effect of redundancy in our data we additionally performed FLYA runs where we added single or multiple additional data types to limited sets of spectra (Fig. 6, columns 9–32 and 43–54), in addition to combinations of 4D NOESY types (columns 55-60). For each ‘basis set’ we focused on the spectra that work well on large proteins, i.e. combinations of HNCO, HNCA and ^15^N-resolved NOESY for backbone assignments and ^13^C-NOESY data for methyl assignments. We observed that the benefit of additional data types depends on the composition of the basis set, but a similar trend is visible. Single amino acid type labeling and, to a lesser extent, unlabeling data improves backbone assignments. In contrast, the topology data have little or even a negative effect on backbone assignments and we see a similar trend in methyl assignments. When reducing the amount of methyl NOESY data no synergies between the scalar coupling and topology data were observed (columns 43–54). However, we found a strong synergy by adding the amide anchored data (columns 33–42). This synergy becomes especially clear when combining methyl-to-amide and methyl-to-methyl 4D NOESYs (columns 55–60). The methyl-to-amide 4D NOESY data benefits most from adding amide-anchored data, but already without these data 50% of the manual assignments can be assigned correctly, as opposed to 77% when including them. Presumably, the methyl-to-amide NOESY data can cluster methyl groups according to shared amide connections, but are unable to unambiguously assign them without backbone assignments or methyl-to-methyl NOESYs.

Despite the fact that each additional data type, i.e. single amino acid type labeling, unlabeling, and topology, has a different effect on assignments, combining these data types creates strong synergies. With only ^15^N-resolved NOESY in the basis set we found synergies between all additional data types so that the overall assignment was better than expected from the individual contributions (columns 15–20). For the HNCO + HNCA basis set, we only observed this synergy when all three data types were combined (column 14). The effect of adding topology data is remarkable as they had a negative influence when used without the other data types. These synergies disappear when the ^15^N-resolved NOESY is combined with HNCO + HNCA data (columns 21–26), or when HNCB and HN(CA)CO spectra are included (columns 27–32).

## Discussion

Obtaining chemical shift assignments for α-helical membrane proteins is challenging in general. The large size of the protein-detergent/lipid complex results in a high overall correlation time and often coincides with internal dynamics, unusual peak shapes, duplicated peaks, limited amide back-exchange when perdeuterated, low stability at the required high temperatures, and the presence of strong peaks from flexible moieties that tend to cover other weak peaks. Altogether this leads to poor spectral quality and a lack of correlations in through-bond and NOESY spectra. One of the currently used approaches to address this problem is to perform selective methyl labeling in combination with single site mutations for assignment (Kofuku et al. 2012, 2014; Solt et al. 2017; Eddy et al. 2018; Xu et al. 2019). However, this approach is time-consuming and often ambiguous as mutations may lead to chemical shift changes in other residues. In this work, we have introduced an alternative assignment procedure that enables to obtain assignments by compensating for poor spectral quality in the less-sensitive triple-resonance NMR experiments and the related loss of many correlations.

Our strategy mainly relies on a combination of backbone scalar couplings and methyl- and amide-anchored NOESY data, which is supplemented by additional data from specific atom unlabeling, single amino acid type ^15^N labeling and water/lipid accessibility. These additional data types were measured using sensitive experiments such as HSQC/TROSY, HNCO, HNCA, and 3D ^13^C- or ^15^N-resolved NOESY. Although the additional data were redundant in the complete dataset, they did contribute significantly when amino acid identifying information, i.e. C^α^/C^β^ correlations, was removed. In addition, we observed a synergy between all of the additional data types, in particular in situations of sparse data. We also observed a strong synergy between the amide-anchored data and 4D NOESY data, indicating that ^15^N labeling is still worthwhile when measuring high molecular weight (membrane) proteins. 4D NOESY spectra were significantly more helpful than 3D NOESYs. We suspect that the low signal dispersion in the NOESY spectra is the main reason for this.

Nearly complete assignments of 7-TM proteins have so far only been obtained for the phototaxis receptor sensory rhodopsin II (pSRII) in DHPC micelles (Gautier et al. 2008, 2010) and for proteorhodopsin (PR) in diC_7_PC micelles (Reckel et al. 2011). For pSRII, the complete backbone and the majority of the side chains could be assigned (Gautier et al. 2008, 2010), whereas for PR backbone assignment was nearly complete (including Hα) and of the side chains only methyl groups were assigned using selective labeling (Reckel et al. 2011). In comparison, both of these systems are smaller due to the use of detergents and also no intermediate conformational exchange or peak doubling, which make assignments of bR much more challenging, has been reported for those cases.

For at least 17% of the bR backbone we failed to observe amide signals, which constitutes about half of the unassigned residues. These are mainly residues from the extracellular side and the majority of helix E. Most of the detected but unassigned spin systems show low signal intensity, lack sequential contacts and NOE connections, or are in overlapping regions, thereby precluding unambiguous assignment. We were able to link some sequential spin systems, but they could not be mapped onto the sequence. Two pairs that could be identified by FLYA were assigned to helix E, close to the retinal ionone ring, but were too ambiguous to use for the assignment. The extracellular loops are a lot smaller than the intracellular loops and therefore less flexible. We hypothe-size the presence of intermediate conformational exchange in the extracellular loops, possibly related to the transport of water into the extracellular side of the helix bundle, and of related motions of aromatic side chains that significantly reduce signal-to-noise.

For GPCRs, intermediate conformational exchange has been claimed to be the biggest problem for NMR spectroscopy (Zerbe 2011). Thermostabilized receptors are in development, which display increased stability by locking the protein into a single state (Serrano-Vega et al. 2008; Tate and Schertler 2009; Lebon et al. 2011; Scott and Plückthun 2013). Their reduced conformational exchange is expected to be beneficial to the overall spectral quality. Of course, in such systems interesting dynamics are largely removed. However, assignments obtained for such a system can still be very useful when removing stabilizing mutations on a one-by-one basis while keeping track of the remaining assignments. When applying our assignment protocol to GPCRs, one is also faced with the problem of back-exchanging amide deuterons to protons. In principle, cell-free expression in light water using perdeuterated amino acids is capable of producing proteins that have protonated amides and deuterated sidechains. Unfortunately, problems of back-exchange of α-protons still remain at present, but we expect that successful protocols to achieve this task will become available soon.

For correctly predicting expected peaks of NOESY spectra our procedure needs a protein structure or (homology) model, and those generally present a unique conformation that lacks dynamics. Extracting NOE contacts from flexible regions can be challenging because the simultaneous presence of different side chain rotamers can lead to multiple contacts (or even none when the side chain is too flexible). Indeed, most methyl groups that we had to assign manually are located on the outside of the helix bundle. To include the effects of dynamics in our analysis we increased the distance cutoff for generating the 4D NOESY expected peaks, but found that the amount of expected contacts increased dramatically, making the assignment even more ambiguous (Fig. S17). Another solution to this problem might be to extract average distances from MD simulations so that dynamic parts are better represented.

Automated assignment methods similar to ours have been proposed, but they are either limited in the amount of data that can be incorporated or not applicable to large molecular weight proteins. The Exner lab used only 4D NOESY data in combination with a known structure (Trautwein et al. 2016). In their procedure intense peaks are considered first, and the resulting unambiguous assignments are taken to the next round. After three iterations, assignments with single and multiple possibilities are obtained. The program MAGMA uses only 4D NOESY data and graph theory to obtain “error-free” assignments of methyl groups, yielding 100% accuracy in a benchmark test of soluble proteins (Pritišanac et al. 2017). However, they claim that on average 3.2 methyl–methyl contacts per methyl group are necessary for reliable assignments, while for bR we observed only 2 contacts on average. Prestegard et al. developed a procedure for sparsely labeled proteins for which no sequential correlation spectra can be measured, i.e. if only methyl or single-amino-acid-type labeled proteins are at hand (Gao et al. 2017). The method predicts HSQC and RDC data from a structure and utilizes a genetic algorithm to match predictions to the measurements. Recently, the method has been expanded to include predictions based on a MD simulation (Chalmers et al. 2019). Programs for backbone-specific automated assignment such as Autoassign (Zimmerman et al. 1997), I-PINE (Lee et al. 2019), MARS (Jung and Zweckstetter 2004), and J-UNIO (Serrano et al. 2012) are limited to good-quality spectra with sufficient sequential correlations. They cannot use 4D NOESY data, but do not need a structure for the assignment unless combined with RDC data. It is the strength of our procedure that it greatly benefits from combining amide and methyl anchored data and includes many unconventional data types.

bR has often been considered as the “ubiquitin of membrane proteins” due to its ease of expression, purification and stability during measurement. Indeed, data for bR have been published decades back, but this study revealed that a major portion of bR is actually invisible for NMR spectroscopy and the peak doubling indicates the presence of two species corresponding to the dark-adaptation, i.e. retinal isomerization in darkness leading to a resting state that cannot be converted directly into the active state by illumination. Interestingly, the intermediate conformational exchange present in the extracellular part of bR is something that is often observed for GPCRs, suggesting that bR still remains an interesting target for further research.

Finally we would like to add a few sentences describing what type of samples and what type of experiments were really worth the effort. Certainly, perdeuteration to the highest achievable degree was crucial, and amide-anchored triple-resonance experiments that transfer magnetization only to neighboring positions were usually of sufficient quality, as was the HNCACB. Given the importance of these experiments for obtaining assignments it is clear that refolding allowed to quantitatively back-exchange all amide deuterons, contributing to the large extend of amide assignments. The latter in turn allowed for the assignment of the majority of methyl groups. We also recommend making the stereospecifically-labeled ILV-labeled species because they (i) added methyl information to the ^15^N NOESY strips, (ii) added helpful unlabeling information without the need to make additional samples, (iii) allowed their assignments from both ^13^C/^15^N and ^13^C/^13^C 4D NOESY data, and (iv) introduce the possibility for stereo-selective labeling, which increases NOESY signal intensities without significantly reducing the amount of cross peaks. From all the alternative data only the topological restraints from water and lipid proximity offered no real benefit for assignments, however, no additional sample nor any additional NMR experiments are necessary to detect water proximity. Finally, single amino acid labeling required producing a number of samples, which, however, were fairly inexpensive and quickly done, and greatly helped in the assignment.

## Electronic supplementary material

Below is the link to the electronic supplementary material.

Supplementary material 1 (DOCX 4757 kb)

## Data Availability

Chemical shifts are available in the BioMagResBank database under the accession number 50009.
