# Supplementary material for: Backbone and methyl assignment of bacteriorhodopsin incorporated into nanodiscs
Source: J Biomol NMR. 2019 Nov 21;74(1):45–60. doi: 10.1007/s10858-019-00289-7 (PMC7015963; doi:10.1007/s10858-019-00289-7)
Supplement: Supplementary file 1 — Supplementary material 1 (DOCX 4757 kb) [file 10858_2019_289_MOESM1_ESM.docx]

**Supplementary Information**

Supplementary Materials & Methods 2

Table S1 NMR experiments 6

Table S2 bR samples and labeling types 7

Table S3 Observed backbone chemical shifts 7

Table S4 Chemical shifts for residues of the minor state in the C-terminus 8

Table S5 Chemical shift differences between duplicated peaks 8

Table S6 Observed NOE cross peaks 9

Figure S7 bO & bR purification 10

Figure S8 Single amino acid labeling [^15^N,^1^H-]TROSY spectra 10

Figure S9 Correlation time - TRACT 11

Figure S10 Peak doubling 12

Figure S11 4D NOESY contact analysis 13

Figure S12 Unlabeling data 14

Figure S13 ^15^N-TROSY spectrum after H/D-exchange 15

Figure S14 Raw FLYA output 15

Figure S15 Location of relatively intense amide signals 16

Figure S16 Proximity data mapped onto the bR reference structure, 1M0L 17

Figure S17 Influence of the increased NOE cutoff on FLYA performance 18

# Supplementary Materials & Methods

*Expression of bO*

The plasmid pET28-misticT-bOp for the mistic-bacterioopsin (mistic-bO) fusion protein was transformed into competent *E. coli* BL21 (DE3) cells. A preculture from a single colony was grown in 5 ml of M9 media (3 g/L KH_2_PO_4_, 6 g/L Na_2_HPO_4_, 0.5 g/L NaCl, 2 mM MgSO_4_, 1 ml trace metal solution, 50 mg/L kanamycin, 1 g/L ^15^NH_4_Cl, 2–3 g/L deuterated ^13^C-glucose) for several hours. To adapt the cells to D_2_O, 5 ml of M9 D_2_O media were inoculated to an OD 0.05 and grown over night at 37 °C. Then, the cells were diluted into M9 D_2_O media to an OD of 0.1 and the doubling time (around 2 h) was checked during the day. An appropriate amount of M9 D_2_O medium was inoculated over night at 37 °C, and the main culture was inoculated the next day to an OD of 0.1–0.2. The cells were grown at 37 °C in 2 l baffled flasks filled with 250 ml of media. One hour before induction (at OD = 0.6) specific precursors were added, otherwise the cells were induced with 0.5 mM of IPTG at an OD = 1, and the temperature was lowered to 18 °C. For uniformly labeling, the cells were grown for 16–20 h. For expression with precursors, the time was reduced to 5–6 h, which resulted in about 50 % lower yields but less scrambling of precursors. The cells were harvested by centrifugation for 20 min at 5000 *g* and stored at –80 °C until further use.

*Precursors for specifically methyl-reversed labeled samples*

*I^δ1^L^δ(R, S)^V^γ(R, S)^:*

For the production of racemic ILV-labeled bacterio-opsin, the following precursors (Cambridge Isotope Laboratories, Inc.) were added: 70 mg alpha-ketobutyric acid, sodium salt (methyl-^13^C, 99%; 3,3-D_2_, 98%) and 160 mg alpha-ketoisovaleric acid, sodium salt (3-methyl-^13^C, 99%; 3,4',4',4'-D_4_, 98%) were added to 1 L medium 1 h prior to the induction.

*A^β^I^δ1^L^δ(R)^V^γ(R)^:*

For the specific labeling of alanine-β, isoleucine-δ1 and stereospecific labeling of leucine-δ(R) and valine-γ(R) methyl groups with the ^13^CH_3_ isotopomer in otherwise uniformly ^2^H, ^13^C, ^15^N-labeled bacterio-opsin, the following precursors (NMR-Bio) were added to the medium according to the manufacturer’s instructions: 3-[^13^C_3_,^1^H]-2-[D]-alanine and 2-hydroxy-2-[2’-^13^C,^1^H -1’-D_2_]ethyl-3-oxo-4[D_3_] butanoic acid, 15–20 min prior to induction and 2-(D_3_) methyl, 2,4-(^13^C_2_,^1^H)-acetolactate, 1h prior to induction.

*A^β^I^δ1^L^δ(R)^V^γ(R)^(U^13^C):*

For the specific labeling of alanine-β, isoleucine-δ_1_ and stereospecific labeling of leucine-δ(R) and valine-γ(R) methyl groups with the ^13^CH_3_ isotopomer in otherwise uniformly ^2^H, ^13^C, ^15^N-labeled bacterio-opsin, the following precursors (NMR-Bio) were added to the medium according to the manufacturer’s instructions: (^13^C_3_)-2-(D)-L-alanine and 2-hydroxy-2-[1’,2’-^13^C_2_-1’-D_2_]ethyl-3-oxo-[1,2,3-^13^C_3_]-4-[D_3_] butanoic acid, 15–20 min prior to induction and 2-(D_3_) methyl (1,2,3,4-^13^C)-acetolactate, 1 h prior to induction.

*A^β^I^γ2^T^γ^:*

For the specific labeling of alanine-β, isoleucine-γ_2_ and threonine-γ methyl groups with the ^13^CH_3_ isotopomer in otherwise uniformly D, ^13^C, ^15^N-labeled bacterio-opsin, the following precursors (NMR-Bio) were added to the medium according to the manufacturer’s instructions: 2-(D)-3-(^13^C)-L-alanine, 2-hydroxy-2-(D_5_)-ethyl-3-oxo-(4-^13^C)-butanoic acid, deuterated α-keto-isovalerate, 2,3-(D_2_),4-(^13^C)-L-threonine and deuterated glycine, 1h prior to induction.

*Methionine:*

For the specific labeling of methionine methyl groups with the ^13^CH_3_ isotopomer, 160 mg/L L-methionine-(methyl-^13^C, 99%) (Sigma) were added 1 h before induction.

*Tryptophan:*

For tryptophan labeling, M9 media was supplemented with 40 mg/L 4,6-dideuterio[5-^13^C]anthranilicacid.

*Single amino acid type labelling*

For the single amino acid labelling, the expression was performed in RF18 cells. The cells were grown in M9-RF18 media, were only one of the essential amino acids was ^15^N labeled. The media was not deuterated, so the main culture was inoculated with an overnight culture to an OD of 0.2. The cells were induced at OD=0.8 with 0.5 mM of IPTG and expressed for 5 h at 18 °C. The M9-RF18 medium contained: 3 g/L KH_2_PO_4_, 7.5 g/L Na_2_HPO_4_ x 2 H_2_O, 0.5 g/L NaCl, 2 mM MgSO_4_, 1 ml trace metal solution, 50 mg/L kanamycin, 1 g/L ^14^NH_4_Cl, 3 g/L ^12^C glucose, 500 mg/L aspartic acid, 230 mg/L isoleucine, 230 mg/L leucine, 230 mg/L valine, 130 mg/L phenylalanine, 170 mg/L tyrosine. The respective ^15^N-labeled amino acid (Sigma) was only added with 100 mg/L.

*Purification of bO*

1–3 g of cells were resuspended in 30 ml of lysis buffer (50 mM Tris pH 8, 100 mM NaCl, 1 mM EDTA, 5 mM MgCl_2_, 1 mg/ml lysozyme, 0.4 mg/ml DNaseI), and were sonicated for 10 min (Branson sonicator, 40 % output, 1 s on, 2 s off). The inclusion bodies were harvested at 40,000 *g* for 40 min at 4 °C and were solubilized overnight in 30 ml of denaturation buffer (20 mM Tris pH 8.4, 150 mM NaCl, 0.25% sarcosyl, 5 mM imidazole, 8 M urea). The solution was cleared for 10 min at 40,000 *g* and the supernatant was loaded with 0.5 ml/min on an Äkta system equipped with two 5 ml HisTrap HP columns. The columns were washed with buffer I (20 mM Tris pH 8.4, 150 mM NaCl, 0.25% sarcosyl) and the protein was eluted with buffer I and 250 mM imidazole. The buffer was exchanged on PD10 columns against buffer I and the fusion protein was cleaved off overnight at room temperature upon addition of 2–10 U thrombin per mg of bO. The next day, 1 mM of PMSF was added and the solution was loaded on two 5 ml HisTrap HP columns and subsequently washed with buffer I, buffer II (20 mM Tris/HCl pH 8, 100 mM NaCl, 0,25 % sarcosyl, 20 mM imidazole) and buffer III (20 mM Tris/HCl pH 8, 50 mM NaCl, 0.2 % SDS). bO was then eluted with buffer III and 150 mM imidazole. The imidazole was removed with PD10 columns equilibrated with buffer III, and the concentration was measured by Nanodrop. The solution was stored at –20 °C until further use. The bO yields were usually 10–20 mg/l M9 D_2_O culture (Fig. S7).

*Expression and purification of MSPΔH5*

MSPΔH5 was expressed for 4 h in BL21 (DE3) cells in TB media at 37°C. 20 g of cells were resuspended in 80 ml Lysis Buffer (50 mM Tris pH 8 at 4 °C, 300 mMNaCl, 15 mM Imidazole, 2 % Triton X-100, 5 mM MgCl_2_, 2 mg/ml Lysozyme, 0.06 mg/ml DNaseI). The Solution was sonicated for 5 min (Branson sonicator, 40 % output, 1 on, 3 s off) on ice. The Lysate was cleared by centrifugation at 35000 g for 30 min and the supernatant was loaded on a ÄKTAprime system at 4 °C equipped with 3 x 5 ml HisTrap HP columns with 0.5 mL/min. The columns were washed with 150 ml of following buffers: Triton Buffer (20 mM Tris pH 8 at 4 °C, 300 mM NaCl, 1 % Triton X-100), Cholate Buffer (20 mM Tris pH 8 at 4°C, 300 mM NaCl, 50 mM Na-Cholate), MSP Buffer (20 mM Tris pH 8 at 4°C, 300 mM NaCl), Wash Buffer (20 mM Tris pH 8 at 4°C, 300 mM NaCl, 50 mM imidazole). The protein was eluted with MSP Buffer and 400 mM imidazole and dialyzed against 2 L of Dialysis buffer (10 mM Tris pH 8, 150 mM NaCl) for 4 h in a 6-8 kDa membrane. The concentration was determined and TEV protease was added in a weight ratio of 1:50, following an overnight dialysis at room temperature against 4 L of Dialysis Buffer with 0.5 mM DTT. The next day, the solution was loaded on 3 × 5 ml HisTrap HP columns and the flow-through, MSP without the His-tag, was collected and dialyzed over night against Nanodisc Buffer (20 mM Tris pH 8, 50 mM NaCl). The concentration was determined by Nanodrop and aliquots with 2–5 mg/ml were stored at –80 °C. The usual yields were 300–600 mg.

*bR reconstitution upon incorporation into nanodiscs*

The scaffold protein MSPΔH5 was expressed and purified as described above. bO was mixed with MSPΔH5, DMPG (25 mM in 2% SDS, 20 mM Tris pH 8) and retinal (10–15 mM in EtOH). The volume was adjusted with nanodisc buffer (20 mM Tris, 50 mM NaCl) for a final bO concentration of 5 µM and a final SDS concentration of 0.5 %. The mixing ratio of bO:MSP:DMPG:retinal was 1:8:400:5. The solution was incubated overnight in the dark at 26 °C. The refolding was induced upon removal of SDS by 0.25 g/ml BioBeads for 2 h at 26 °C. Then another 0.25 g/ml BioBeads were added for an additional hour. The BioBeads were removed by filtration. To remove the excess of empty nanodiscs, the solution was loaded on a 1 ml HisTrap HP column and was washed with nanodisc buffer. bR nanodiscs were eluted with nanodisc buffer and 150 mM imidazole. To remove aggregates and undigested mistic-BR-nanodiscs, the elution was concentrated with an Amicon Ultra 4 with a 50 kDa cut off and purified on a Superdex 200 Increase 10/300 column. The fractions from the main peak were pooled and the buffer was exchanged with a PD10 column to the NMR buffer (40 mM K-Phosphate, pH 6.4, 0.2 mM NaN_3,_ 10% D_2_O). The concentration was measured by Nanodrop and the sample was concentrated to 200 µl with 200­–500 µM, and was filled into a Shigemi NMR tube. The final yield of pure monodisperse bR nanodiscs was about 40 % of the bO used in the reconstitution mixture.

*Deuterium exchange*

The exchange of a bR nanodiscs NMR sample into a pure D_2_O buffer (40 mM K-Phosphate pD 6.4 in 99.8% D_2_O) was done with a Zeba Spin Desalting Column, 7K MWCO, 0.5 ml. The exchange was performed two times to ensure a complete exchange.

# Table S1 NMR experiments

| Sample | Experiment | Field (MHz) | Mixing time (ms) | D1 (s) | NS | SW(F1) (ppm) | SW(F2) (ppm) | SW(F3) (ppm) | O2P(13C) (ppm) | O3P(15N) (ppm) | TD2 (2D F1, 3D F2) | TD3 (3D F1) |
| --- | --- | --- | --- | --- | --- | --- | --- | --- | --- | --- | --- | --- |
| 1 | 2D HSQC 15N | 900 |  | 1.5 | 128 | 34 | 18 |  |  | 118 | 300 |  |
| 1 | 2D HSQC 13C ali ct | 900 |  | 1.5 | 16 | 40 | 13 |  | 20 |  | 400 |  |
| 1 | 3D N15 NOESY | 700 | 200 | 2 | 16 | 10 | 30 | 13.9 |  | 117 | 50 | 127 |
| 1 | 3D HNCO | 700 |  | 1 | 8 | 30 | 30 | 18 | 176 | 117 | 44 | 100 |
| 1 | 3D HNCA | 700 |  | 1 | 16 | 30 | 30 | 18 | 54 | 117 | 44 | 95 |
| 1 | 3D HN(CO)CA | 700 |  | 1 | 24 | 30 | 30 | 18 | 54 | 117 | 44 | 100 |
| 1 | 3D HN(CO)CB | 700 |  | 1 | 64 | 65 | 30 | 18 | 39 | 117 | 40 | 100 |
| 1 | 3D HN(CA)CO | 700 |  | 1.3 | 16 | 15 | 30 | 18 | 176 | 117 | 40 | 100 |
| 2 | 2D HSQC 15N | 700 |  | 1.7 | 256 | 30 | 18 |  |  | 117 | 117 |  |
| 2 | 2D HSQC 13C ali ct | 700 |  | 1 | 32 | 40 | 13.3 |  | 20 |  | 280 |  |
| 2 | 3D 15N NOESY | 700 | 200 | 1 | 16 | 10 | 30 | 13.9 |  | 117 | 44 | 128 |
| 2 | 3D HN(CA)CO | 700 |  | 1.3 | 32 | 15 | 30 | 18 | 178 | 117 | 40 | 76 |
| 2 | 3D HNCB | 700 |  | 1.5 | 64 | 65.3 | 30.1 | 18 | 39 | 117 | 40 | 57 |
| 3 | 2D HSQC 15N | 700 |  | 1.7 | 256 | 30 | 18 |  |  | 117 | 128 |  |
| 3 | 3D HNCO | 700 |  | 1 | 8 | 30 | 30 | 18 | 176 | 117 | 44 | 100 |
| 3 | 3D HNCA | 700 |  | 1 | 16 | 30 | 30 | 18 | 54 | 117 | 44 | 100 |
| 4 | 2D HSQC 15N | 700 |  | 1.5 | 192 | 30 | 18 |  |  | 117 | 200 |  |
| 4 | 3D HNCO | 700 |  | 1 | 8 | 15 | 30 | 18 | 176 | 117 | 44 | 80 |
| 8 | 2D HSQC 13C ali ct | 700 |  | 1 | 32 | 40 | 13.3 |  | 20 |  | 280 |  |
| 8 | 3D C13 NOESY | 700 | 200 | 1.2 | 32 | 11 | 30 | 13.7 | 16 | 117 | 44 | 110 |
| 9-13 | 2D HSQC ^15^N - best | 700 |  | 0.2 | 1536 | 35 | 16.23 |  |  | 117 | 150 |  |
|  |  | **Field (MHz)** | **Mixing time (ms)** | **D1 (s)** | **NS** | **t1 (ms)** | **t2 (ms)** | **t3 (ms)** | **t4 (ms)** | **NUS (%)** | **Processed Points** (F4*F3*F2*F1) |  |
| 5 | 4D 13C-15N NOESY | 950 | 250 | 0.7 | 16 | 16.1 | 14 | 17.6 | 33.6 | 11.1 | 512*88*128*80 |  |
| 5 | 4D 13C-13C NOESY | 900 | 250 | 0.5 | 4 | 17.1 | 22.5 | 22.5 | 66.5 | 11.6 | 512*192*192*80 |  |
| 6 | 3D HN(CA)CB | 800 |  | 0.7 | 16 | 9 | 31.3 | 53.2 |  |  |  |  |
| 6 | 3D HN(COCA)CB | 800 |  | 0.6 | 32 | 5.2 | 29.9 | 53.2 |  |  |  |  |
| 6 | 3D C13 NOESY (hC-NH) | 950 | 250 | 0.7 | 16 | 20.9 | 19.2 | 41.5 |  |  |  |  |
| 6 | 3D C13 NOESY (hN-CH) | 950 | 250 | 0.7 | 8 | 22.4 | 36.8 | 73.7 |  |  |  |  |
| 6 | 3D N15-N15 NOESY | 900 | 250 | 0.5 | 32 | 19.5 | 19.7 | 43 |  |  |  |  |
| 7 | 4D 13C-15N NOESY | 950 | 250 | 0.7 | 16 | 15.2 | 13.4 | 17.6 | 33.6 | 17.2 | 512*88*128*64 |  |
| 7 | 4D 13C-13C NOESY | 900 | 250 | 0.5 | 4 | 16.1 | 14.9 | 22.4 | 66.5 | 21 | 512*192*128*64 |  |
| 7 | 3D C13 NOESY (Hc-CH) | 900 | 250 | 0.5 | 16 | 18.6 | 23.2 | 65.5 |  |  |  |  |
| 7 | 3D C13 NOESY (hC-CH) | 900 | 250 | 0.5 | 8 | 23.2 | 23.2 | 65.5 |  |  |  |  |

# Table S2 bR samples and labeling types

| Sample | Labeling | Nanodisc | Protein Labeling | Methyl Labeling | Sidechain Labeling |
| --- | --- | --- | --- | --- | --- |
| 1 | ILV | ^2^H-DMPG | ^15^N/^13^C/^2^H | Leu: δ_1_ + δ_2_  Val: γ_1_ + γ_2_  Ile: δ_1_ | ^13^C/^2^H |
| 2 | Uniform | ^1^H-DMPG | ^15^N/^13^C/^2^H | - | - |
| 3 | AILV | ^2^H-DMPG | ^15^N/^13^C/^2^H | Leu: δ_1_ + δ_2_  Val: γ_1_ + γ_2_  Ile: δ_1_;Ala: β | AIV: ^12^C/^2^H  L: ^13^C/^2^H |
| 4 | AIT | ^2^H-DMPG | ^15^N/^13^C/^2^H | Ala: β  Ile: γ_2_  Thr: γ | AITGV: ^12^C/^2^H |
| 5 | AILV - Stereoselective (proR) | ^2^H-DMPG | ^15^N/^13^C/^2^H | Leu: δ_1_  Val: γ_1_  Ile: δ_1_; Ala: β | AIV: ^12^C/^2^H  L: ^13^C/^2^H |
| 6 | ILV + W(Z_3_) | ^2^H-DMPG | ^15^N/^13^C/^2^H | Leu: δ_1_ + δ_2_  Val: γ_1_ + γ_2_  Ile: δ_1_;Trp: ξ_3_ | ^13^C/^2^H |
| 7 | ILV - Noesy | ^2^H-DMPG | ^15^N/^13^C/^2^H | Leu: δ_1_ + δ_2_  Val: γ_1_ + γ_2_  Ile: δ_1_ | ^12^C/^2^H |
| 8 | MILV | ^2^H-DMPG | ^15^N/^13^C/^2^H | Leu: δ_1_ + δ_2_  Val: γ_1_ + γ_2_  Ile: δ_1_;Met: ε | IV: ^12^C/^2^H  L: ^13^C/^2^H  M: ^12^C/^1^H |
| 9 | Ile ^15^N/ ^1^H | ^1^H-DMPG | ^14^N/^12^C/^1^H |  | - |
| 10 | Leu^15^N/ ^1^H | ^1^H-DMPG | ^14^N/^12^C/^1^H |  | - |
| 11 | Val ^15^N/ ^1^H | ^1^H-DMPG | ^14^N/^12^C/^1^H |  | - |
| 12 | Phe^15^N/ ^1^H | ^1^H-DMPG | ^14^N/^12^C/^1^H |  | - |
| 13 | Tyr ^15^N/ ^1^H | ^1^H-DMPG | ^14^N/^12^C/^1^H |  | - |

# Table S3 Observed backbone chemical shifts

Count of observed chemical shifts derived from experiments relying on scalar couplings for the amide-anchored spin systems. The number of observed C’–chemical shifts denotes the amount of spin systems.

| Atom | Observed | Assigned |
| --- | --- | --- |
| CA | 207 | 155 |
| CA_–1_ | 182 | 143 |
| CB | 169 | 132 |
| CB_–1_ | 125 | 106 |
| C’_–1_ | 210 (83%)* | 156 (62% / 74%)** |
| C’ | 143 | 121 |
| All 6 scalar couplings | 82 | 73 |
| Containing 1 sequential contact | 72 | 38 |
| Containing 0 sequential contacts | 16 | 5 |

* Percentage over the total number of residues ; ** Percentage over total number of residues and observed spin systems, respectively

# Table S4 Chemical shifts for residues of the minor state in the C-terminus

Chemical shifts of the minor state observed in the C-terminus including the difference from the major state in brackets.

| Residue | H | N | C' | CA | CB |
| --- | --- | --- | --- | --- | --- |
| E234 | 8.26 (-0.07) | 120.22 (-0.38) | 174.86 (+0.84) | 55.82 (-0.12) | 29.06 (+0.21) |
| A235 | 7.88 (+0.30) | 124.29 (+1.91) | 175.54 (-0.13) | 49.62 (+0.22) | 18.70 (-0.44) |
| P236 |  |  | 175.87 (+0.77) | 62.16 (+0.23) | 33.27 (-2.25) |
| E237 | 8.47 (-0.27) | 122.47 (-0.88) | 174.86 (+0.04) | 54.34 (-0.48) | 28.30 (+0.28) |
| P238 |  |  | 176.32 (+0.74) | 62.22 (+0.82) | 33.34 (-2.32) |
| S239 | 8.40 (-0.17) | 116.41 (-1.17) | 174.45 (+0.00) | 57.92 (-0.14) | 63.16 (-0.02) |
| A240 | 8.26 (-0.09) | 125.21 (+0.29) | N.D. | N.D. | N.D. |
| G241 | 8.13 (+0.02) | 107.72 (-0.25) | 173.02 (+1.03) | 44.86 (+0.06) |  |
| D242 | 7.79 (+0.30) | 123.71 (-3.54) | 179.23 (-2.25) | 54.34 (-0.32) | 40.20 (+0.14) |
| G243 |  |  |  |  |  |
| A244 |  |  |  |  |  |
| A245 |  |  |  |  |  |
| A246 | 8.05 (-0.01) | 123.58 (-1.20) | 176.98 (+1.08) | 52.09 (+0.00) | 17.97 (-0.01) |
| T247 | 7.57 (+0.32) | 118.32 (-6.34) | N.D. | 62.51 (-1.00) | 70.30 (-1.23) |

# Table S5 Chemical shift differences between duplicated peaks

Chemical shift difference of peak doubling given for individual atoms. The difference is given in absolute numbers, because the major and minor states could not be distinguished.

| Res. | ΔH (ppm) | ΔN (ppm) | ΔC-1 (ppm) | ΔC (ppm) | ΔCA (ppm) | ΔCB (ppm) |
| --- | --- | --- | --- | --- | --- | --- |
| G16 | 0.04 | 0.1 | 0.0 | 0.1 | 0.1 | N.D. |
| M20 | 0.03 | 0.0 | 0.1 | 0.0 | 0.1 | 0.1 |
| G21 | 0.01 | 0.1 | 0.0 | N.D. | 0.0 | N.D. |
| G23 | 0.04 | 0.0 | 0.1 | N.D. | 0.0 | N.D. |
| D36 | 0.03 | 0.2 | 0.0 | N.D. | 0.0 | 0.0 |
| A51 | 0.02 | 0.4 | 0.0 | 0.0 | 0.0 | 0.0 |
| L92 | 0.06 | 0.3 | 0.1 | 0.1 | 0.1 | 0.1 |
| L94 | 0.03 | 0.3 | 0.0 | N.D. | 0.0 | 0.1 |
| L97 | 0.08 | 0.2 | 0.0 | N.D. | 0.0 | 0.1 |
| A98 | 0.10 | 0.1 | 0.1 | 0.1 | 0.1 | 0.1 |
| L99 | 0.01 | 0.2 | 0.0 | 0.1 | 0.0 | N.D. |
| L100 | 0.03 | 0.2 | 0.0 | N.D. | 0.0 | 0.2 |
| V101 | 0.08 | 0.1 | 0.1 | N.D. | 0.0 | 0.1 |
| A103 | 0.08 | 0.1 | 0.0 | 0.0 | 0.0 | 0.3 |
| V112 | 0.02 | 0.4 | 0.0 | 0.0 | 0.0 | 0.2 |
| G113 | 0.06 | 0.0 | N.D. | N.D. | 0.0 | N.D. |
| A114 | 0.05 | 0.0 | 0.0 | 0.1 | 0.0 | 0.1 |
| D115 | 0.07 | 0.5 | 0.1 | N.D. | 0.1 | 0.5 |
| G116 | 0.01 | 0.2 | 0.0 | 0.0 | 0.0 | N.D. |
| G120 | 0.03 | 0.1 | 0.0 | 0.0 | 0.0 | N.D. |
| F153 | 0.03 | 0.4 | 0.3 | N.D. | N.D. | 0.0 |
| F154 | 0.08 | 0.1 | 0.5 | N.D. | 0.2 | N.D. |
| G155 | 0.02 | 0.2 | 0.0 | 0.1 | 0.0 | N.D. |
| S158 | 0.12 | 0.3 | 0.0 | N.D. | 0.0 | 0.0 |
| K159 | 0.08 | 0.1 | 0.1 | 0.0 | N.D. | 0.0 |
| F171 | 0.04 | 0.1 | 0.0 | 0.0 | 0.0 | 0.0 |
| R175 | 0.05 | 0.0 | 0.0 | N.D. | 0.1 | 0.0 |
| V179 | 0.03 | 0.2 | 0.0 | N.D. | 0.1 | 0.2 |
| L181 | 0.07 | 0.1 | 0.1 | N.D. | 0.1 | 0.1 |
| W182 | 0.03 | 0.0 | 0.1 | N.D. | N.D. | 0.3 |
| A184 | 0.05 | 0.0 | 0.1 | 0.1 | 0.0 | 0.1 |
| L201 | 0.04 | 0.1 | 0.0 | N.D. | N.D. | N.D. |
| D212 | 0.04 | 0.3 | 0.1 | N.D. | 0.1 | 0.0 |
| V213 | 0.02 | 0.3 | 0.1 | N.D. | 0.0 | 0.0 |
| F219 | 0.06 | 0.2 | 0.2 | N.D. | 0.1 | 0.0 |
| G220 | 0.04 | 0.1 | 0.0 | N.D. | 0.0 | N.D. |
| F230 | 0.07 | 0.3 | 0.0 | N.D. | 0.0 | 0.1 |

# Table S6 Observed NOE cross peaks

Number of observed NOE cross-peaks per NOESY spectrum. The NOESY types are classified according to their anchors (last two capital letters). Small letters indicate spins correlated by NOEs. When not specified, the labeling pattern of ILV-ProR/S is used (sample 7, Table S2)

|  | Spectrum | NOE peaks, excluding anchors | Spectrometer field (MHz) |
| --- | --- | --- | --- |
| 1 | H-NH (amide-amide) | 478 | 700 |
| 2 | hN-NH | 374 | 900 |
| 3 | H-NH (methyl-amide) | 187 | 700 |
| 4 | Hc-CH | 150 | 900 |
| 5 | hC-CH | 111 | 900 |
| 6 | hC-NH | 146 | 950 |
| 7 | hN-CH | 102 | 950 |
| 8 | 4D HC-CH | 100 | 900 |
| 9 | 4D HC-CH (AILV-ProR) | 146 | 900 |
| 10 | 4D HC-NH | 164 | 950 |
| 11 | 4D HC-NH (AILV-ProR) | 230 | 950 |


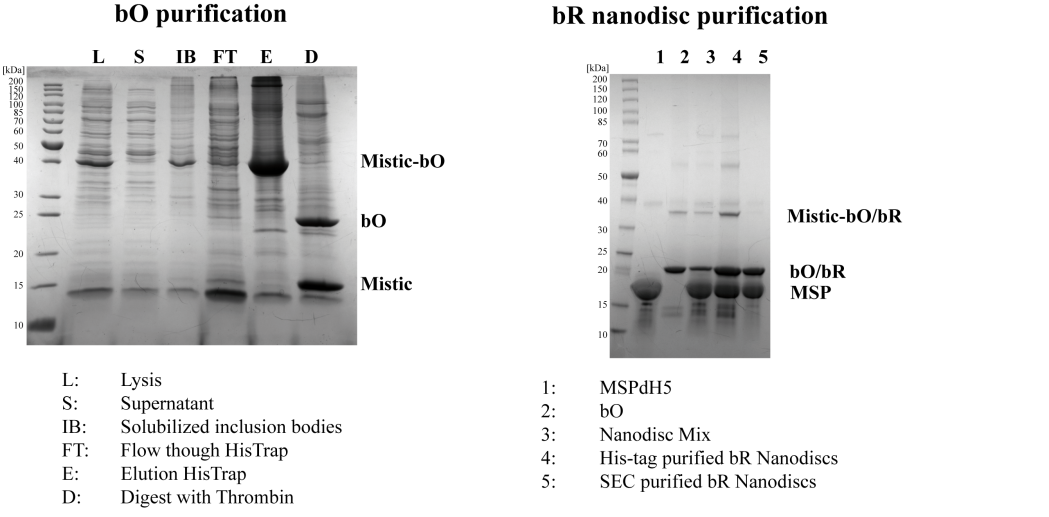


# Figure S7 bO & bR purification

SDS page gels from bO (left) and bR (right) purification steps


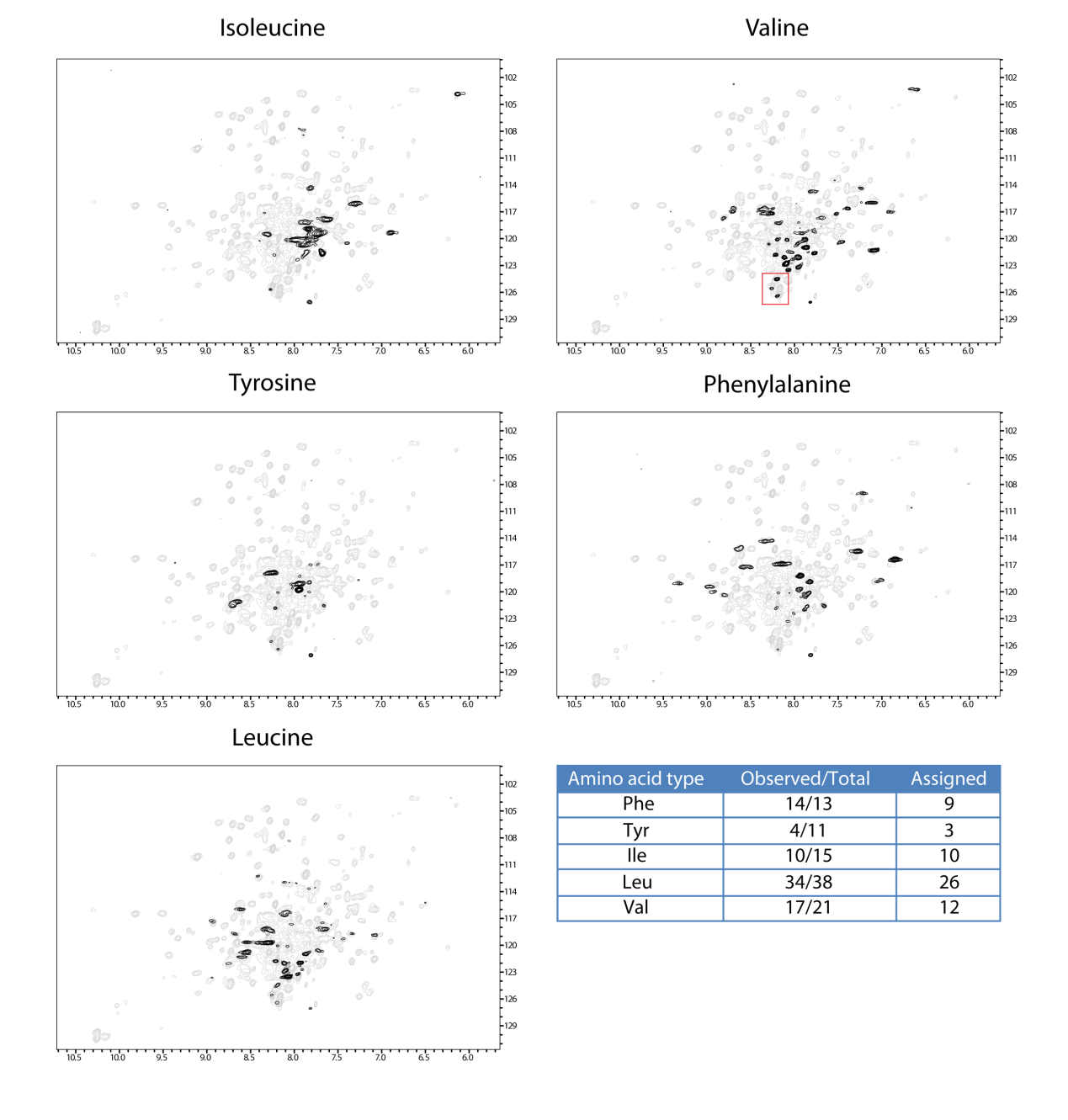


# Figure S8 Single amino acid labeling [^15^N,^1^H-]TROSY spectra

^15^N-TROSY spectra of single amino acid ^15^N-labeled samples (black) with the uniformly labeled spectrum in the background (grey). The signals in the red square in the valine spectrum show the amount of scrambling during expression. Scrambling is observed only in the C-terminus where the peak intensities are ~5 times higher than in the rest of the spectrum.


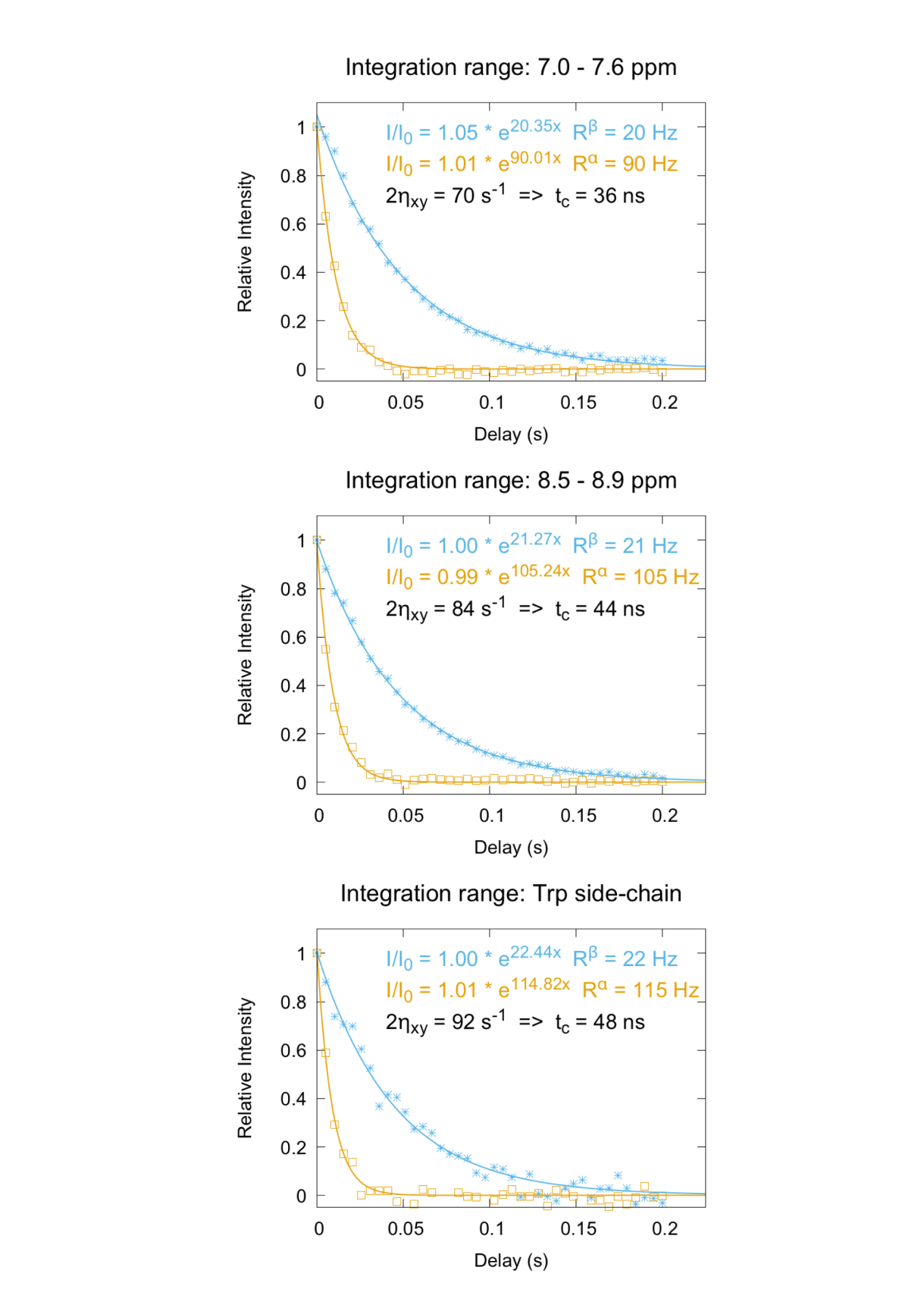


# Figure S9 Correlation time - TRACT

Determination of the correlation time by TRACT experiments. Different integration ranges of the 1D-spectrum were used to avoid the influence of slowly relaxing signals. The measurements were done on a 700 MHz spectrometer at 320 K. Correlation times range from 36 to 44 ns. As the TRACT experiment is sensitive to slowly relaxing signals, e.g. signals from the loops, we estimate the correlation time for bR to be 44 ns.


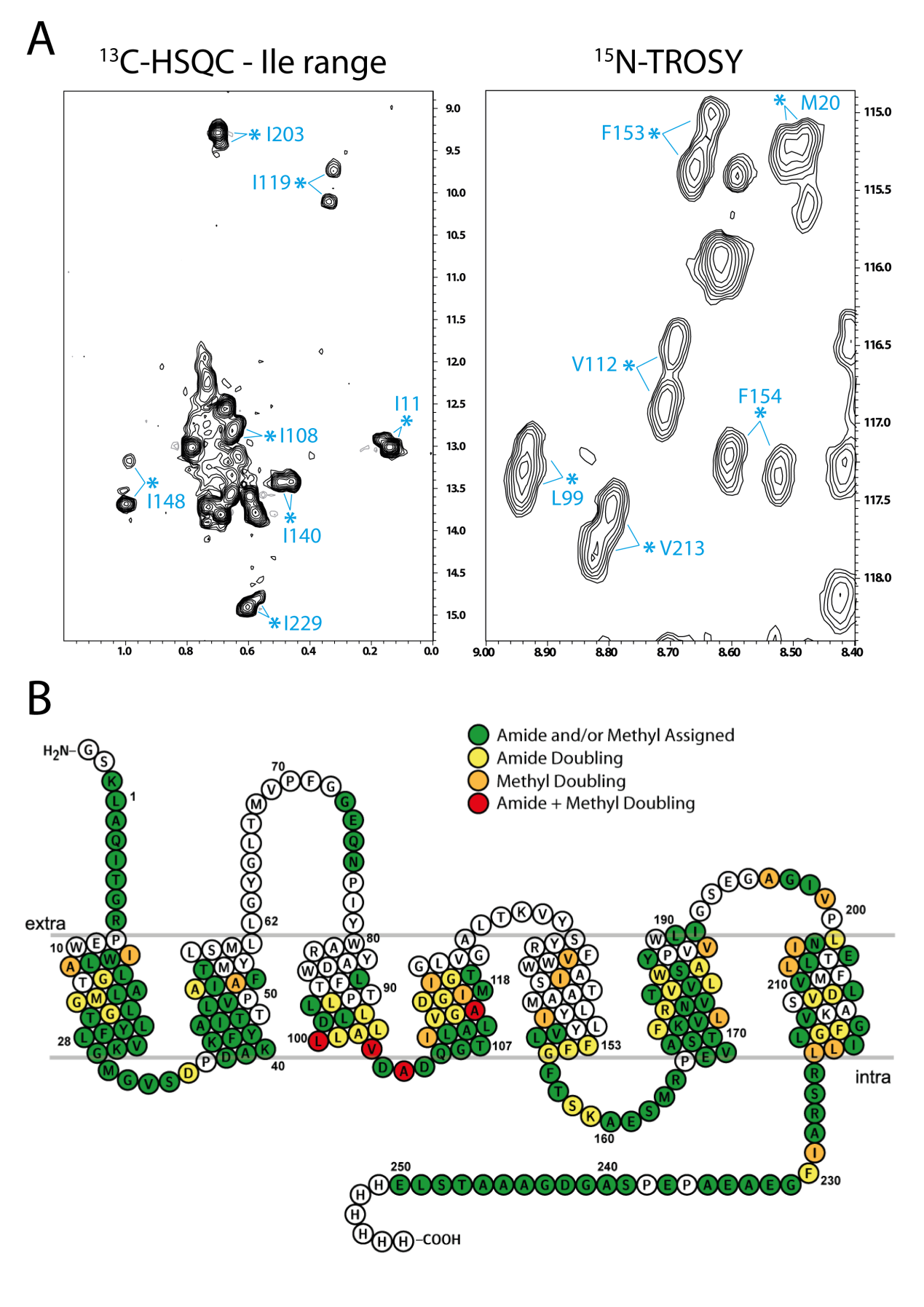


# Figure S10 Peak doubling

(A) Peak doubling as a result of dark-adaptation in the ^13^C-HSQC (left) and ^15^N-TROSY (right). Blue asterisks mark the peak doubles. (B) Extent of doubling as observed in amide and methyl signals.

#
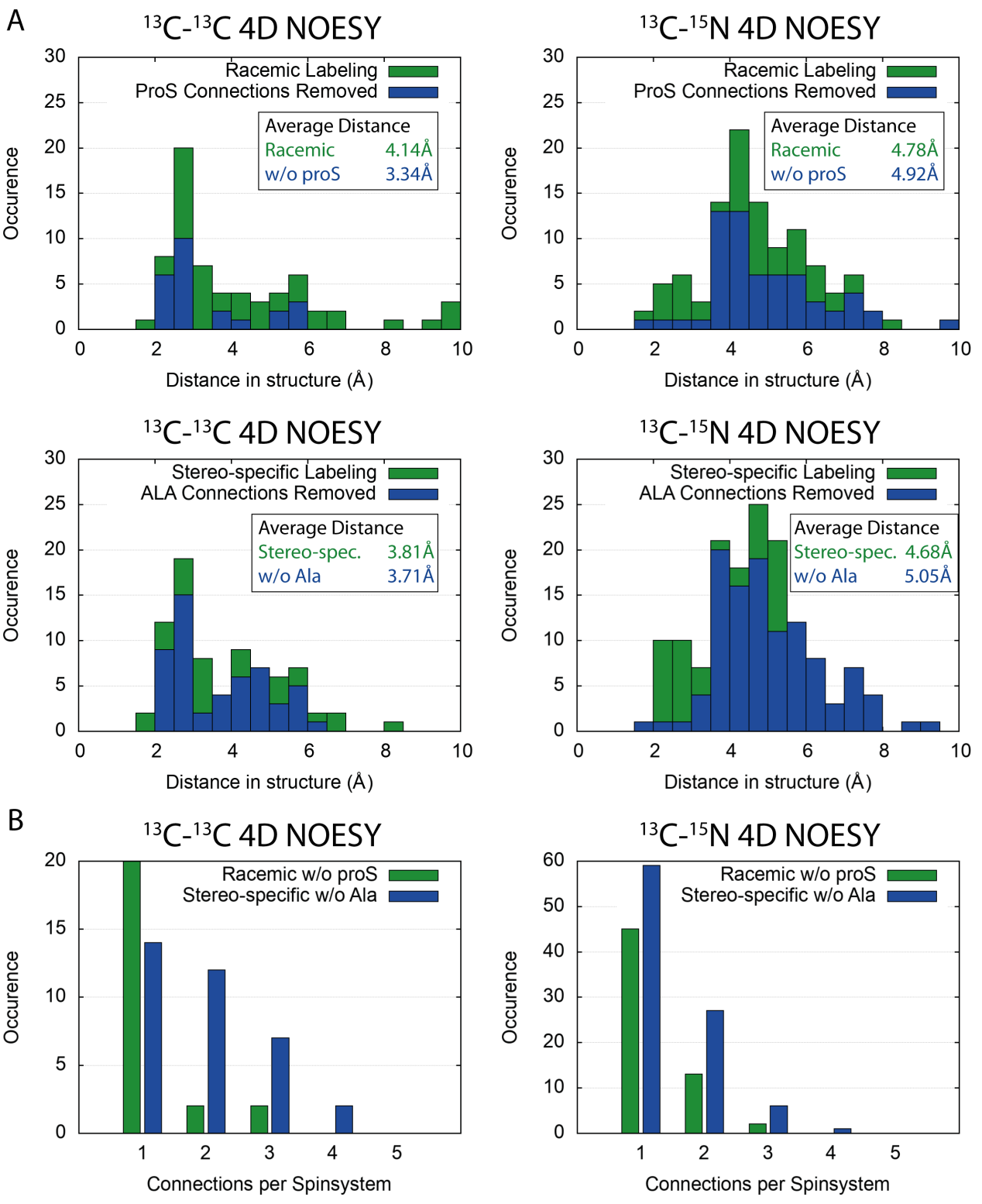
Figure S11 4D NOESY contact analysis

(A) Histogram of closest methyl proton distance extracted from the 1M0L crystal structure for each individual NOE connection for which both the anchor and cross-peak have been assigned. All connections over 8.5 Å are overestimated due to side chain flexibility that is not reflected in the crystal structure. Two NOESY-type spectra were measured, methyl-to-methyl and methyl-to-amide, with two different labeling types, ILV (proR+proS) and AILV (proR). To compare both labeling types, proS connections from ILV and alanine connections from AILV labeling were removed, effectively keeping only the ILV(proR) connections from both labeling types.  The inset shows the average distance over all connections. (B) Histogram of the number of observed NOE connections per spin system, comparing only the ILV(proR) connections.


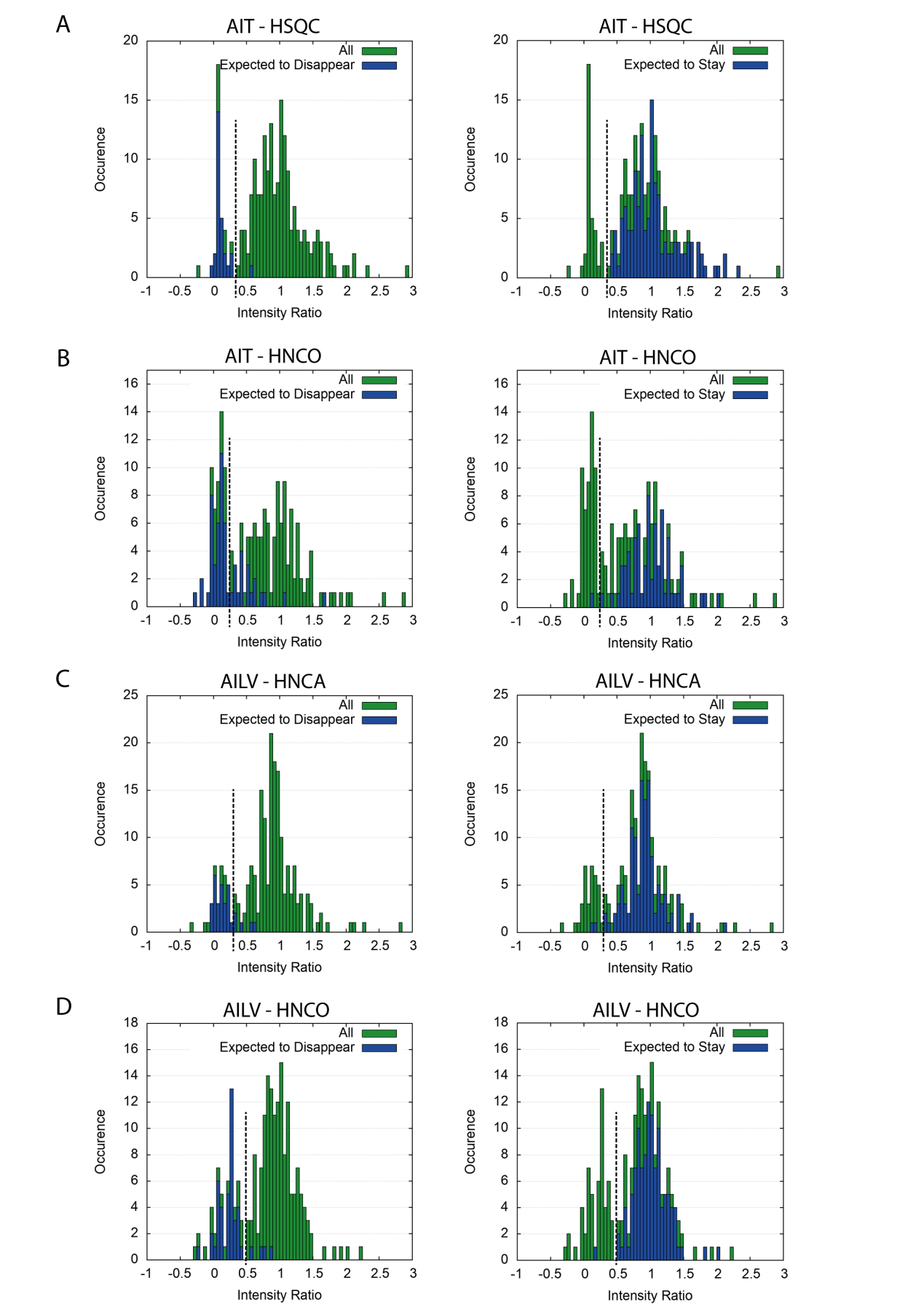


# Figure S12 Unlabeling data

Histograms of the peak intensity ratio of the limited labeled sample (AIT/AILV) versus full ^13^C labeling (ILV) for: (A) [^1^H,^15^N]-TROSY with AIT labeling, (B) HNCO with AIT labeling, (C) HNCA with AILV labeling and (D) HNCO with AILV labeling. The threshold used to identify disappeared peaks is indicated with a dashed line. The spin systems that were identified in (A) were removed from the analysis for (B). The ratio for all spin systems is shown in green, the spin systems that are assigned and expected to disappear (left) or stay (right) are overlaid in blue.


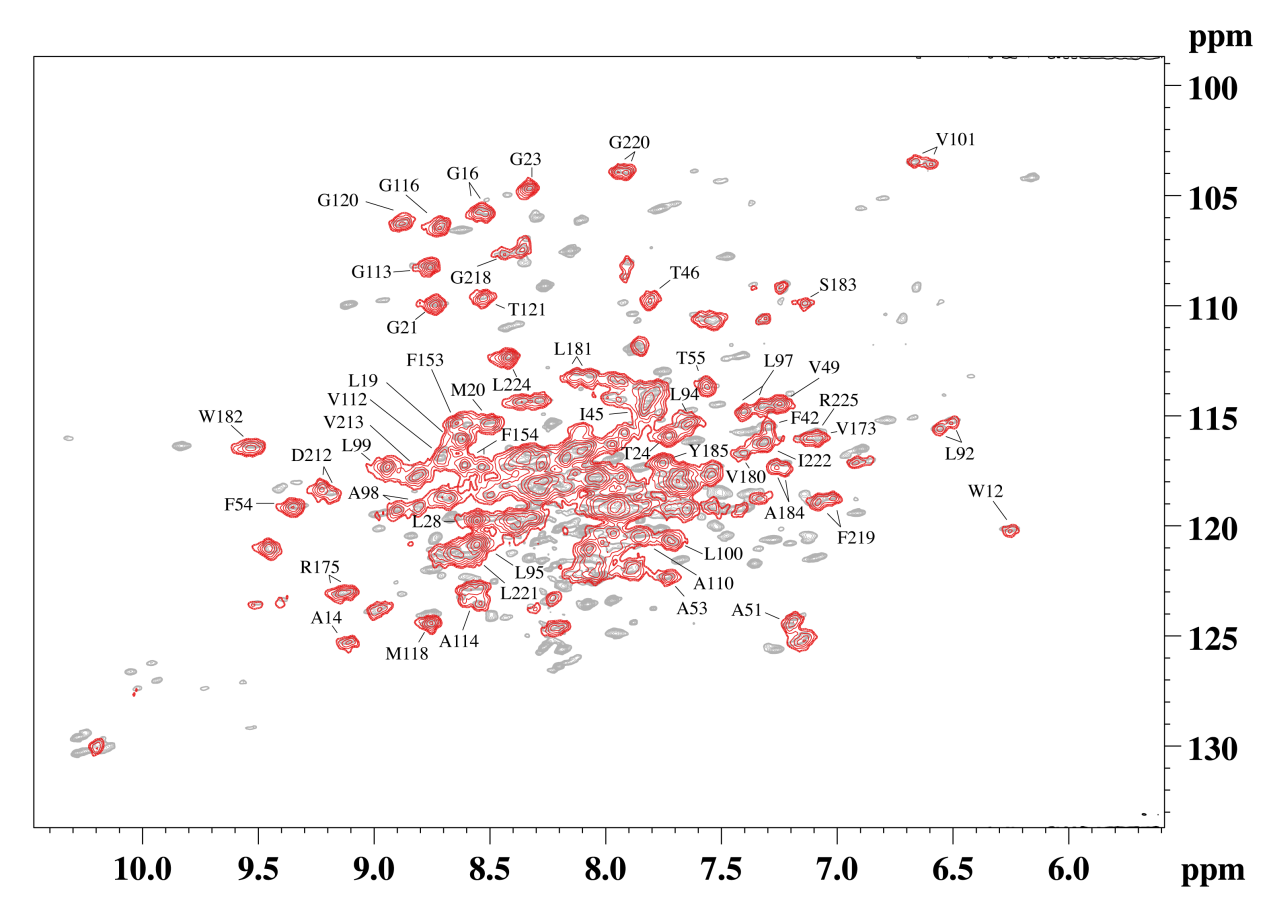


# Figure S13 ^15^N-TROSY spectrum after H/D-exchange

Overlay of bR measured in H_2_O (grey) and D_2_O (red). Measurement was done ~4 hours after transfer into D_2_O. Assigned signals that remained have been annotated, with the exception of the central region. No significant differences were observed after 24 hours in D_2_O.


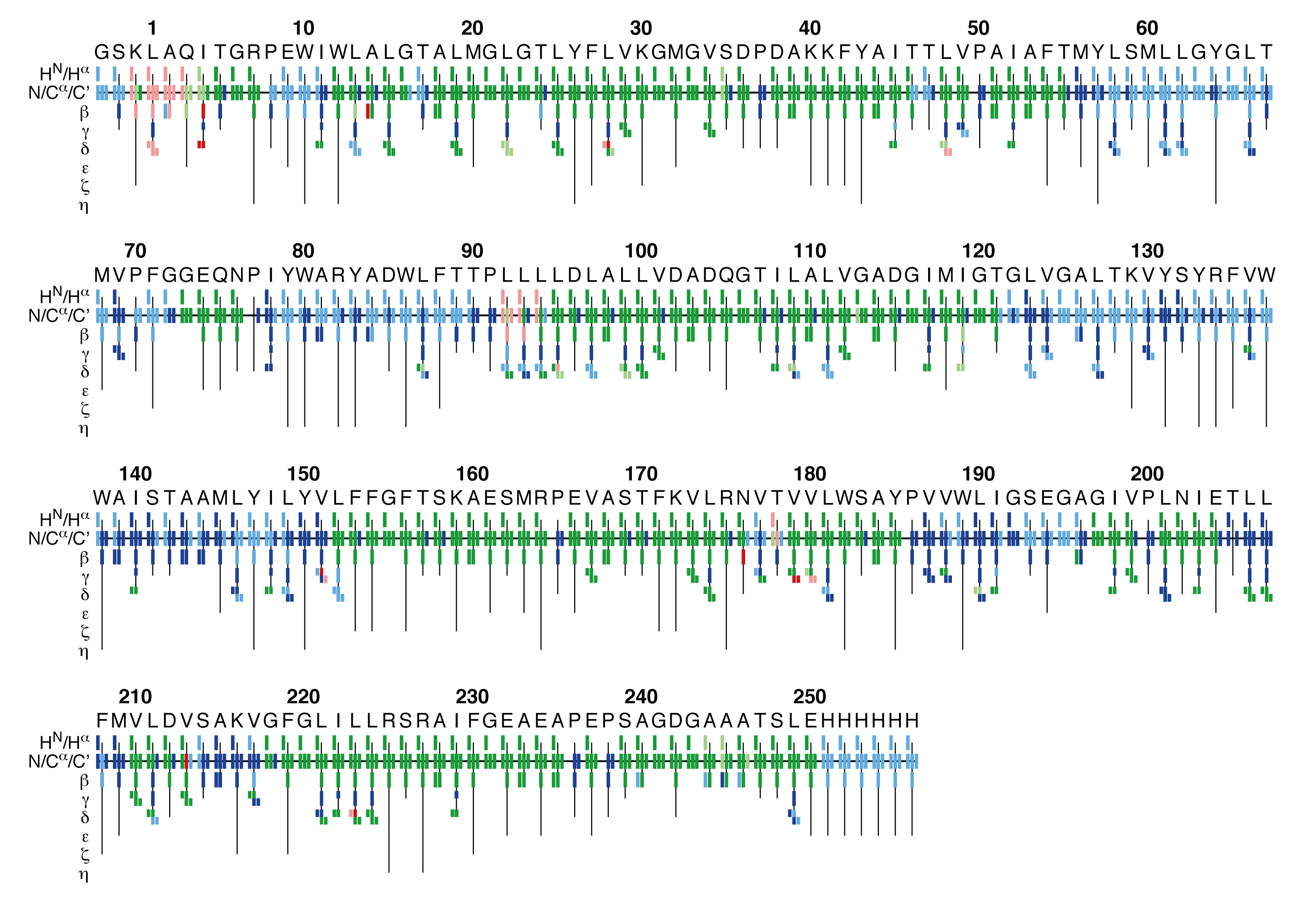


# Figure S14 Raw FLYA output

FLYA assignments obtained with all data included, and comparison to the final, manually curated assignments. Each assignment for an atom is represented by a rectangle; colored green, if the assignment by FLYA agrees with the manually determined reference chemical shifts within a tolerance of 0.03 ppm for ^1^H or 0.4 ppm for ^13^C/^15^N; red, if the assignment differs; blue, if assigned by FLYA but no manual assignment is available. Dark and light colors represent ‘strong’ (self-consistent) and ‘weak’ (tentative) assignments, resp., as classified by chemical shift consolidation from 40 independent runs of the assignment algorithm. An assignment is classified as strong if 80% or more of the runs yielded, within tolerance, the same chemical shift value. The row labeled H^N^/H^α^ shows for each residue H^N^ on the left. The N/C^α^/C’ row shows for each residue the N, C^α^, and C’ assignments from left to right. The rows β-η show the side chain assignments for the heavy atoms in the center and hydrogen atoms to the left and right. For branched side chains, the corresponding row is split into an upper part for one branch and a lower part for the other branch.


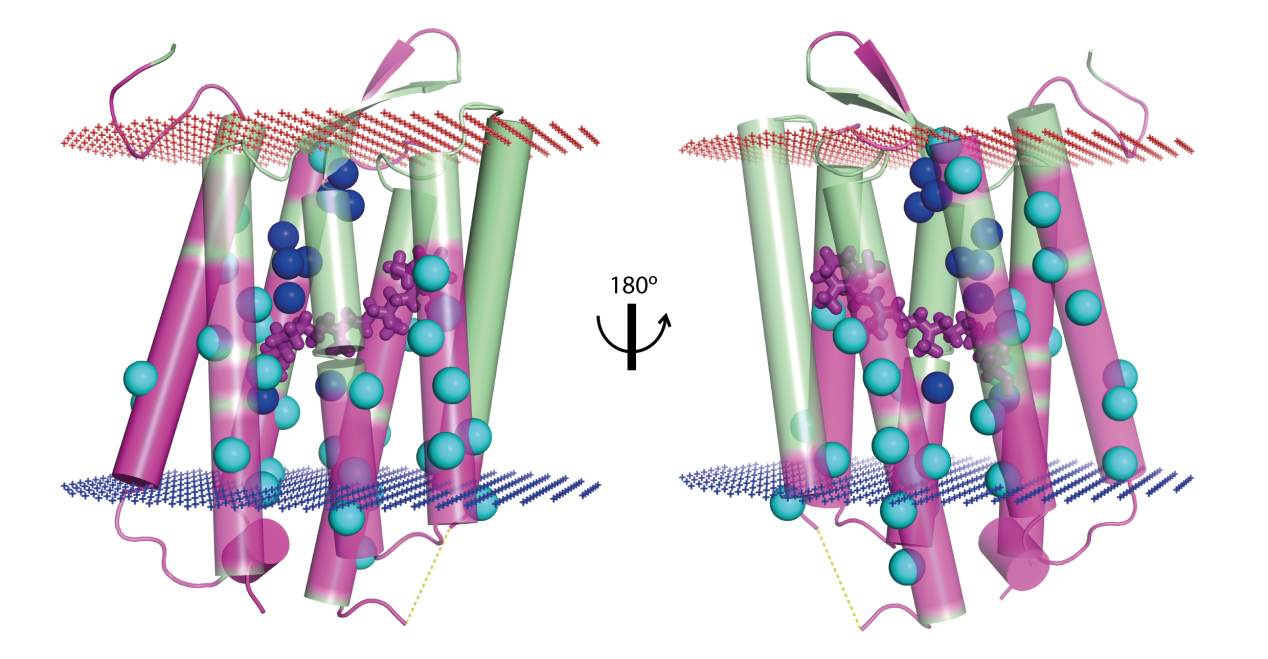


# Figure S15 Location of relatively intense amide signals

Mapping of intense amide signals, as shown in Figure 5a with triangles, as cyan spheres onto the reference structure, 1M0L. Assigned/unassigned backbone is colored in magenta/green. The retinal is shown as purple sticks and internal water molecules as dark blue spheres. The predicted membrane-water interface is indicated by red (extracellular) and blue (intracellular) crosses.


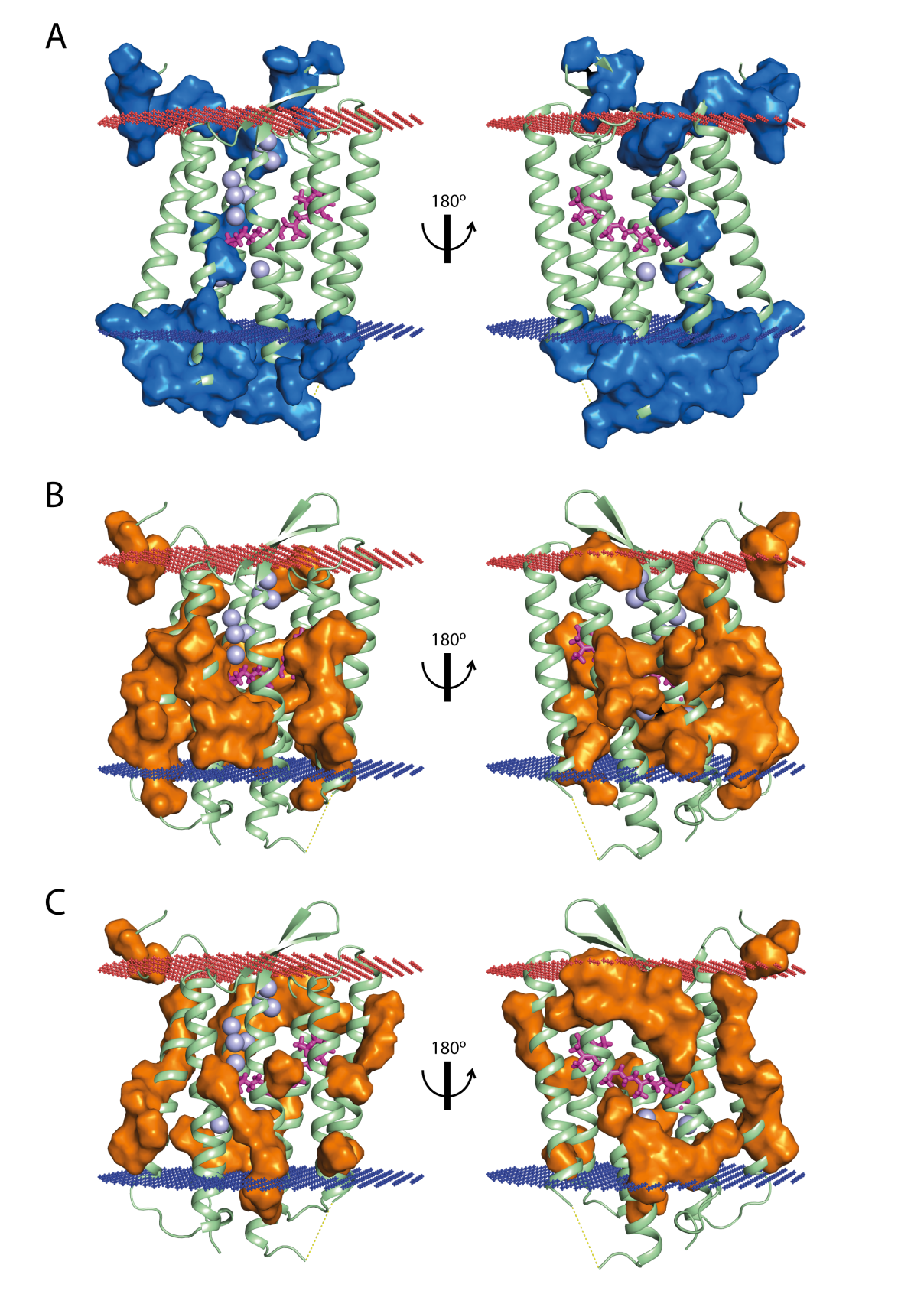


# Figure S16 Proximity data mapped onto the bR reference structure, 1M0L

Whole residues are shown in surface representation for amide-to-water proximity (blue), (B) amide-to-lipid proximity (orange), and (C) methyl-to-lipid proximity (orange). The retinal is shown as purple sticks and internal water molecules as light blue spheres. The predicted membrane-water interface is indicated by red (extracellular) and blue (intracellular) crosses.


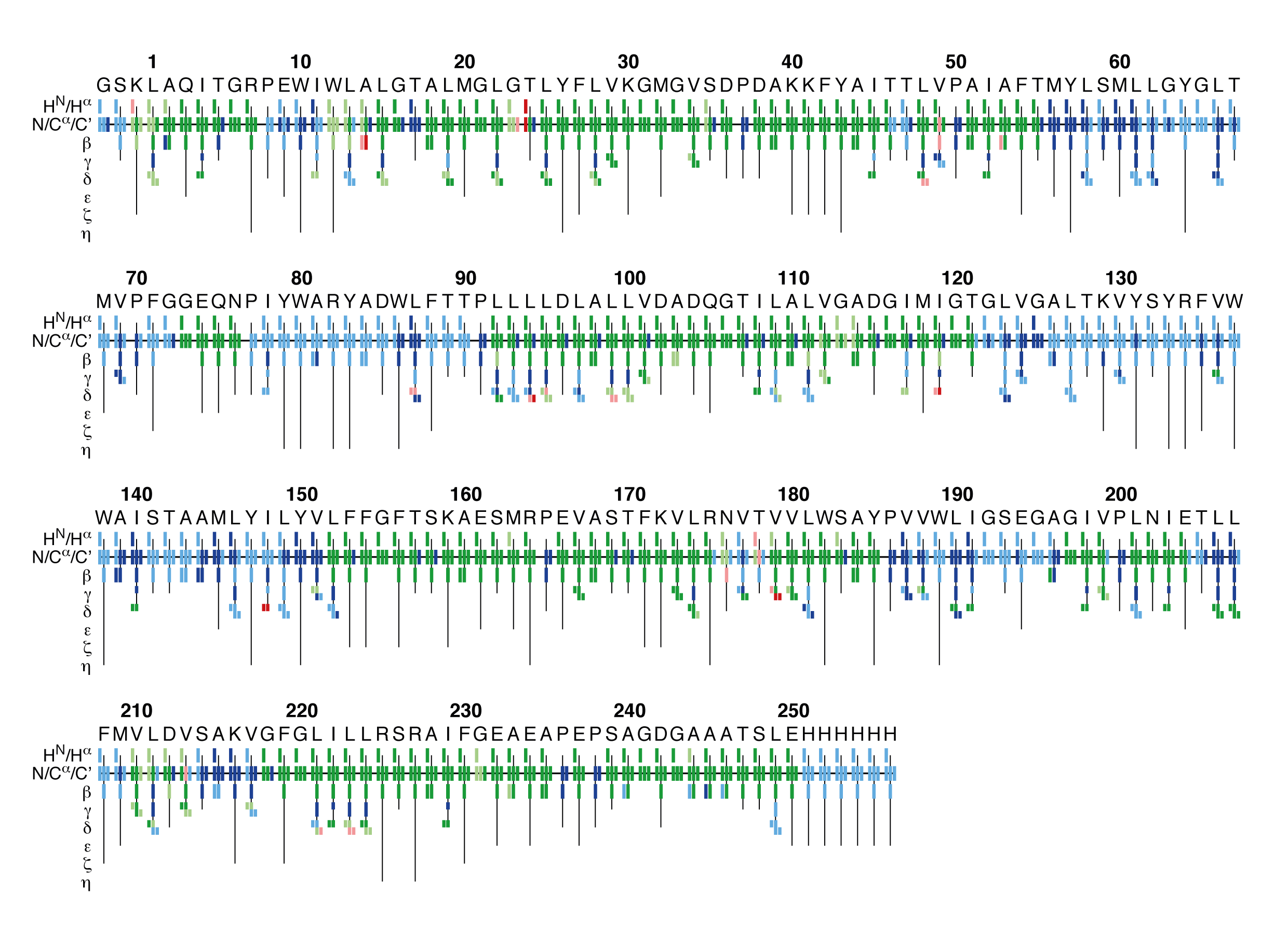


# Figure S17 Influence of the increased NOE cutoff on FLYA performance

FLYA assignments obtained under the same conditions as in Fig. S14, except that the distance cutoff for expected peak generation for all 4D NOESYs was raised from 4.5 to 7 Å. See Fig. S14 for details.
